# Supplementary material for: Verrucosidin Derivatives from the Deep Sea Cold-Seep-Derived Fungus Penicillium polonicum CS-252
Source: Int J Mol Sci. 2022 May 16;23(10):5567. doi: 10.3390/ijms23105567 (PMC9144169; doi:10.3390/ijms23105567)
Supplement: Supplementary file 1 [file ijms-23-05567-s001.zip › ijms-1697526-supplementary.pdf]

Supplementary File

for

Unusual Verrucosidin Derivatives from the Deep Sea Cold-Seep-derived Fungus *Penicillium polonicum* CS-252

Yan-He Li [1,2,3], Xiao-Ming Li [1,2], Xin Li [1,2], Sui-Qun Yang [1,2], Bin-Gui Wang [1,2,3,4,*], and Hong-Lei Li [1,2,*]

[1] CAS and Shandong Province Key Laboratory of Experimental Marine Biology, Institute of Oceanology, Chinese Academy of Sciences, Nanhai Road 7, Qingdao 266071, People's Republic of China; E-Mails: liyanhe1025@163.com (Y.-H.L.); lixmqdio@126.com (X.-M.L.); lixin871014@163.com (X.L.); suiqunyang@163.com (S.-Q.Y.);

[2] Laboratory of Marine Biology and Biotechnology, Qingdao National Laboratory for Marine Science and Technology, Wenhai Road 1, Qingdao 266237, People's Republic of China

[3] College of Marine Science, University of Chinese Academy of Sciences, Yuquan Road 19A, Beijing 100049, People's Republic of China

[4] Center for Ocean Mega-Science, Chinese Academy of Sciences, Nanhai Road 7, Qingdao 266071, People's Republic of China

* Correspondence: wangbg@ms.qdio.ac.cn (B.-G.W.); lihonglei@qdio.ac.cn (H.-L.L.); Tel.: +86-532-82898553 (B.-G.W.): +86-532-82898890 (H.-L.L.)

Content

Figure S1. HRESIMS spectrum of compound **1**.

Figure S2. ^{1}H-NMR (500 MHz, DMSO-d_6) spectrum of compound **1**.

Figure S3. ^{13}C-NMR (125 MHz, DMSO-d_6) and DEPT spectra of compound **1**.

Figure S4. COSY spectrum of compound **1**.

Figure S5. HSQC spectrum of compound **1**.

Figure S6. HMBC spectrum of compound **1**.

Figure S7. NOESY spectrum of compound **1**.

Figure S8. HRESIMS spectrum of compound **2**.

Figure S9. ^{1}H-NMR (500 MHz, DMSO-d_6) spectrum of compound **2**.

Figure S10. ^{13}C-NMR (125 MHz, DMSO-d_6) and DEPT spectra of compound **2**.

Figure S11. COSY spectrum of compound **2**.

Figure S12. HSQC spectrum of compound **2**.

Figure S13. HMBC spectrum of compound **2**.

Figure S14. NOESY spectrum of compound **2**.

Figure S15. HRESIMS spectrum of compound **3**.

Figure S16. ^{1}H-NMR (500 MHz, DMSO-d_6) spectrum of compound **3**.

Figure S17. ^{13}C-NMR (125 MHz, DMSO-d_6) and DEPT spectra of compound **3**.

Figure S18. COSY spectrum of compound **3**.

Figure S19. HSQC spectrum of compound **3**.

Figure S20. HMBC spectrum of compound **3**.

Figure S21. NOESY spectrum of compound **3**.

Figure S22. HRESIMS spectrum of compound **4**.

Figure S23. ^{1}H-NMR (500 MHz, DMSO-d_6) spectrum of compound **4**.

Figure S24. ^{13}C-NMR (125 MHz, DMSO-d_6) and DEPT spectra of compound **4**.

Figure S25. COSY spectrum of compound **4**.

Figure S26. HSQC spectrum of compound **4**.

Figure S27. HMBC spectrum of compound **4**.

Figure S28. NOESY spectrum of compound **4**.

Figure S29. HRESIMS spectrum of compound **5**.

Figure S30. ^{1}H-NMR (500 MHz, DMSO-d_6) spectrum of compound **5**.

Figure S31. ^{13}C-NMR (125 MHz, DMSO-d_6) and DEPT spectra of compound **5**.

Figure S32. COSY spectrum of compound **5**.

Figure S33. HSQC spectrum of compound **5**.

Figure S34. HMBC spectrum of compound **5**.

Figure S35. HRESIMS spectrum of compound **6**.

Figure S36. ^{1}H-NMR (500 MHz, DMSO-d_6) spectrum of compound **6**.

Figure S37. ^{13}C-NMR (125 MHz, DMSO-d_6) and DEPT spectra of compound **6**.

Figure S38. COSY spectrum of compound **6**.

Figure S39. HSQC spectrum of compound **6**.

Figure S40. HMBC spectrum of compound **6**.

Figure S41. NOESY spectrum of compound **6**.

Figure S42. DP4+ probability Excel sheets of compound **4**.

Figure S43. DP4+ probability Excel sheets of compound **5**.

Figure S1. HRESIMS spectrum of compound **1**.

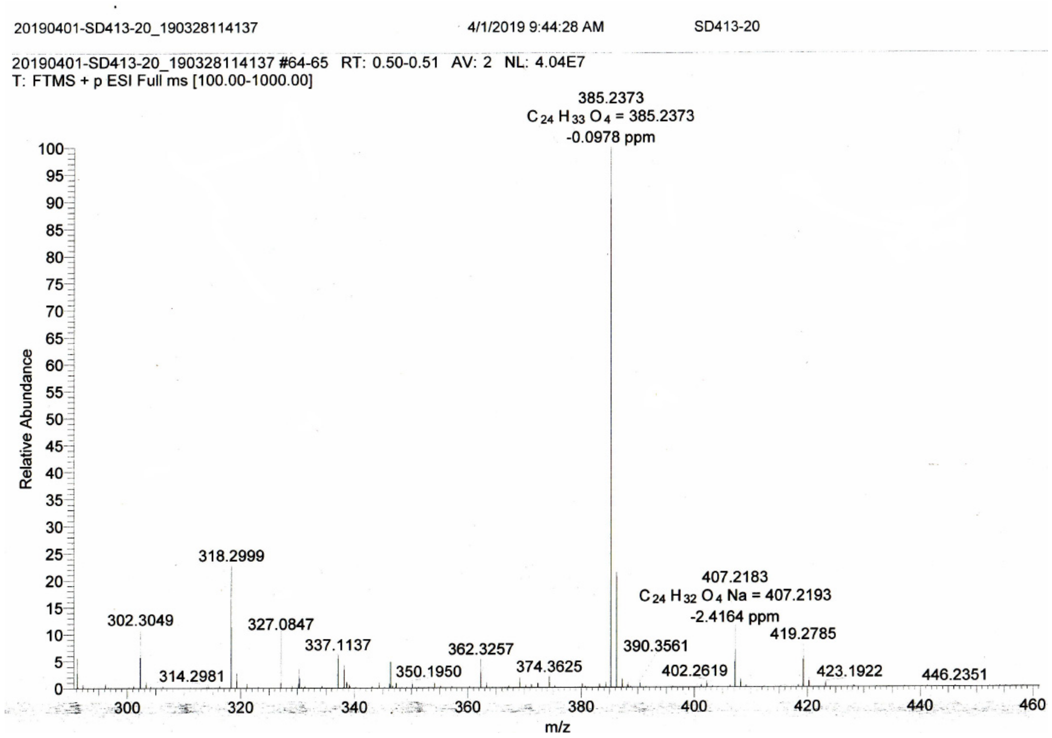

Figure S2. ^{1}H-NMR (500 MHz, DMSO-d_6) spectrum of compound **1**.

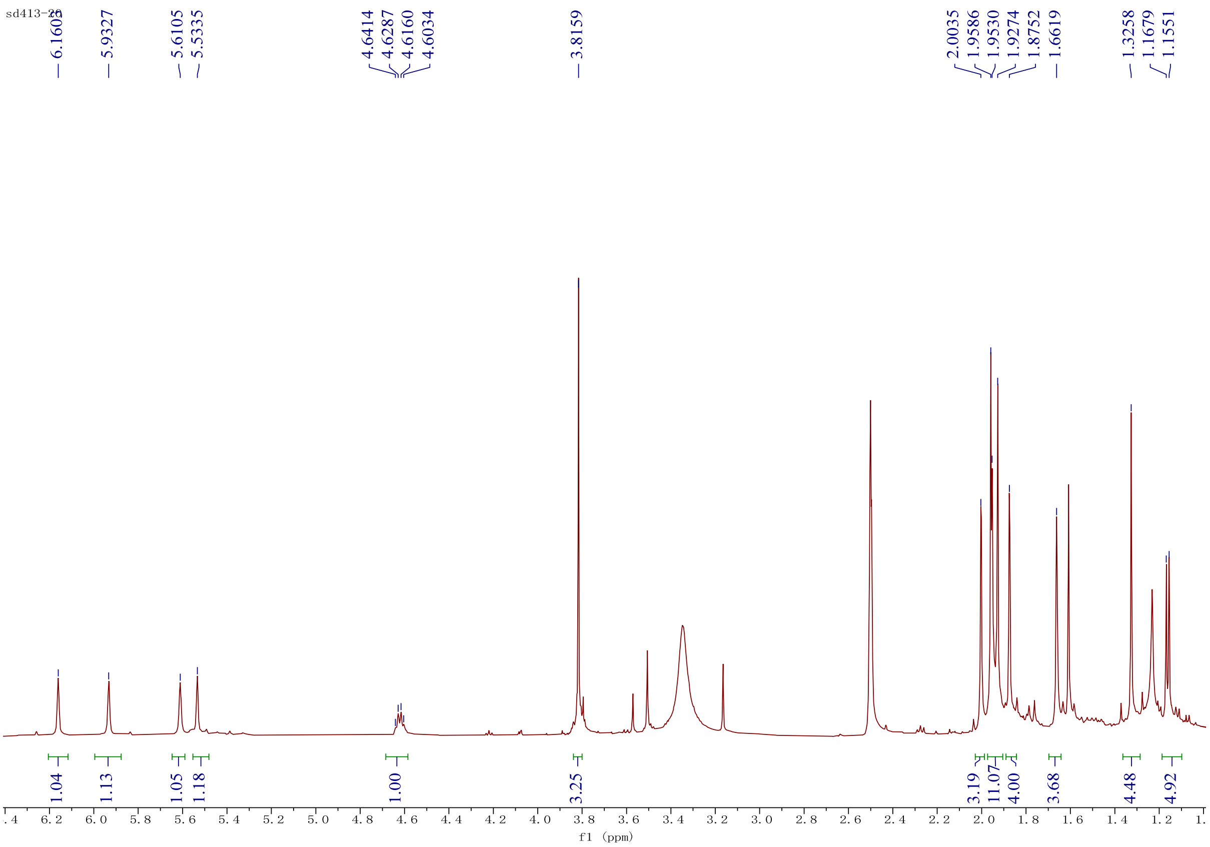

Figure S3. ^{13}C-NMR (125 MHz, DMSO-d_6) and DEPT spectra of compound **1**.

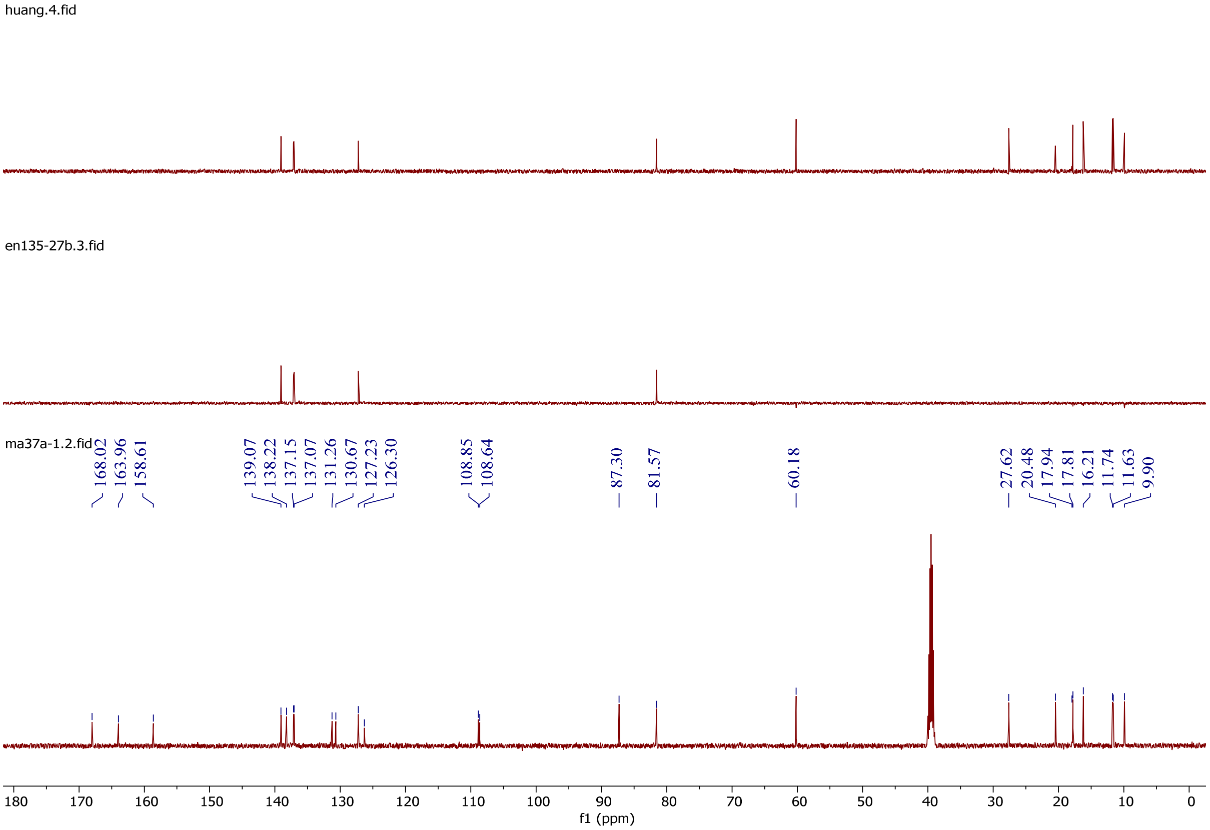

Figure S4. COSY spectrum of compound **1**.

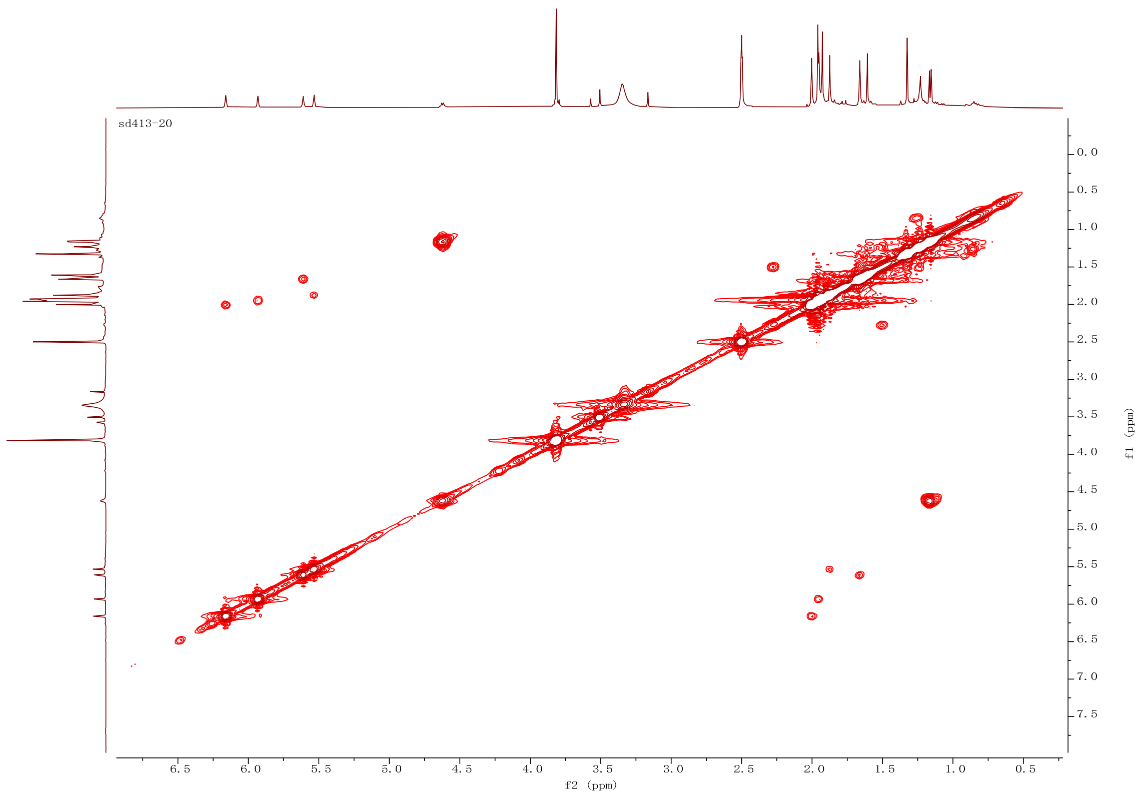

Figure S5. HSQC spectrum of compound **1**.

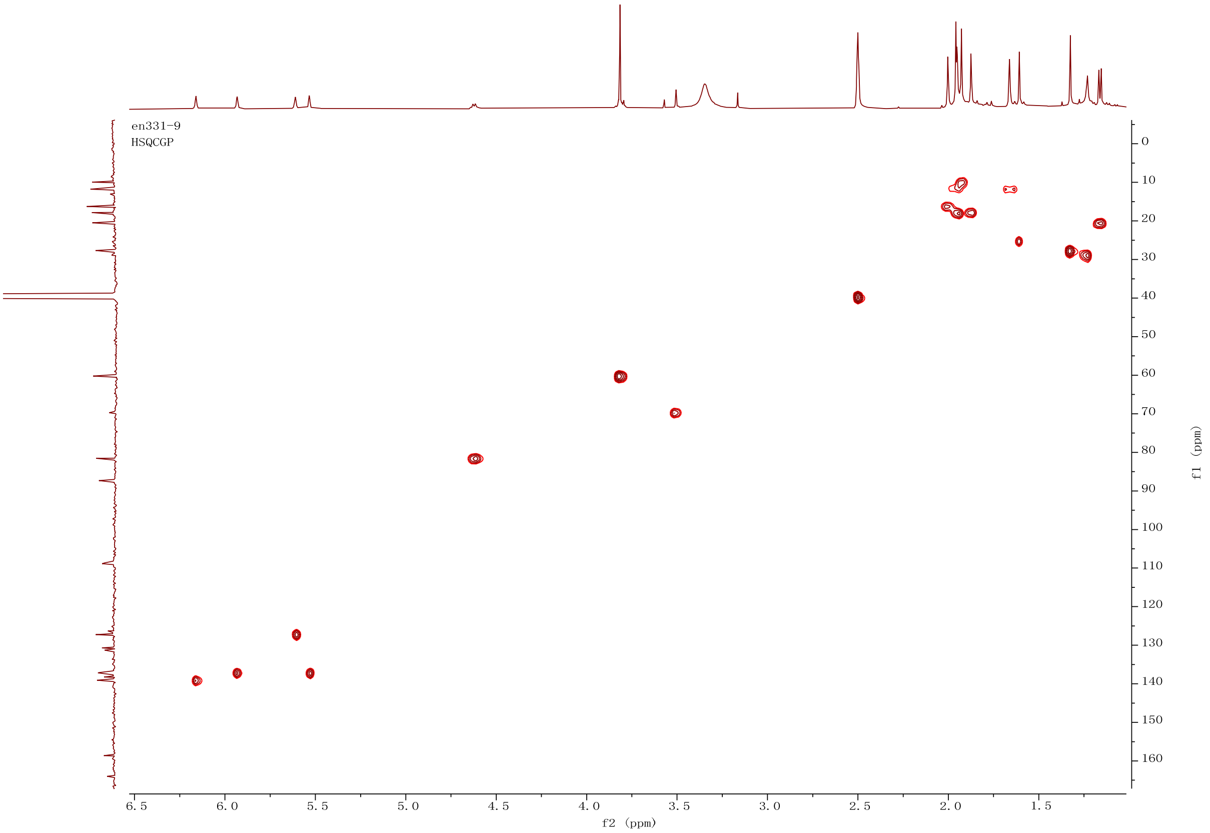

Figure S6. HMBC spectrum of compound **1**.

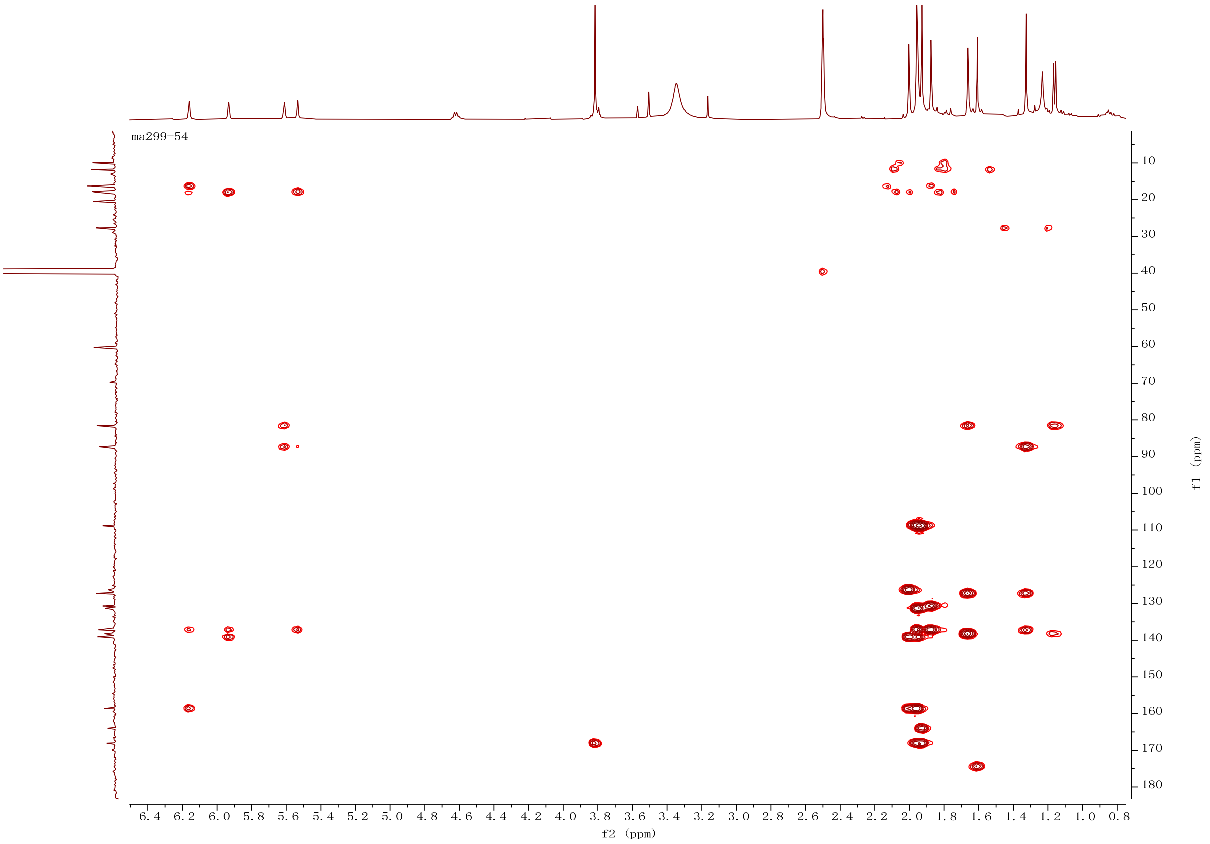

Figure S7. NOESY spectrum of compound **1**.

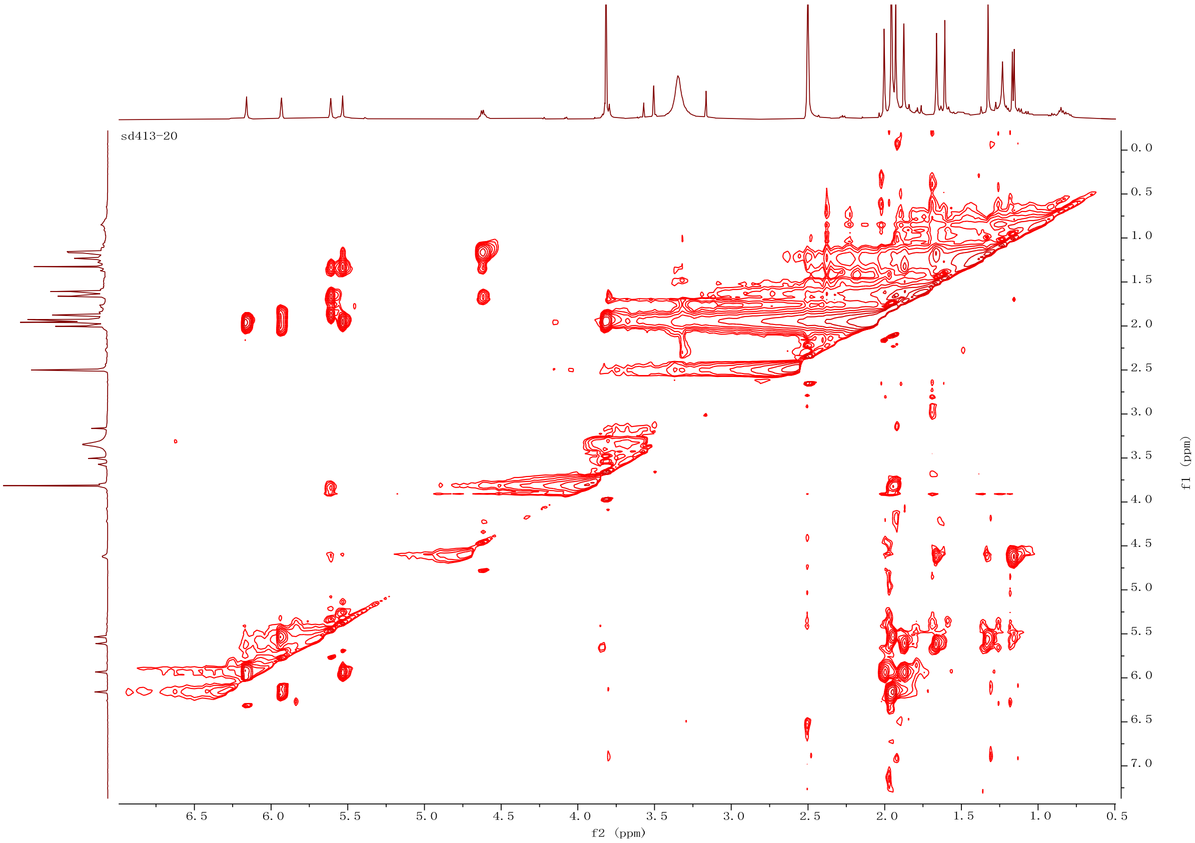

Figure S8. HRESIMS spectrum of compound **2**.

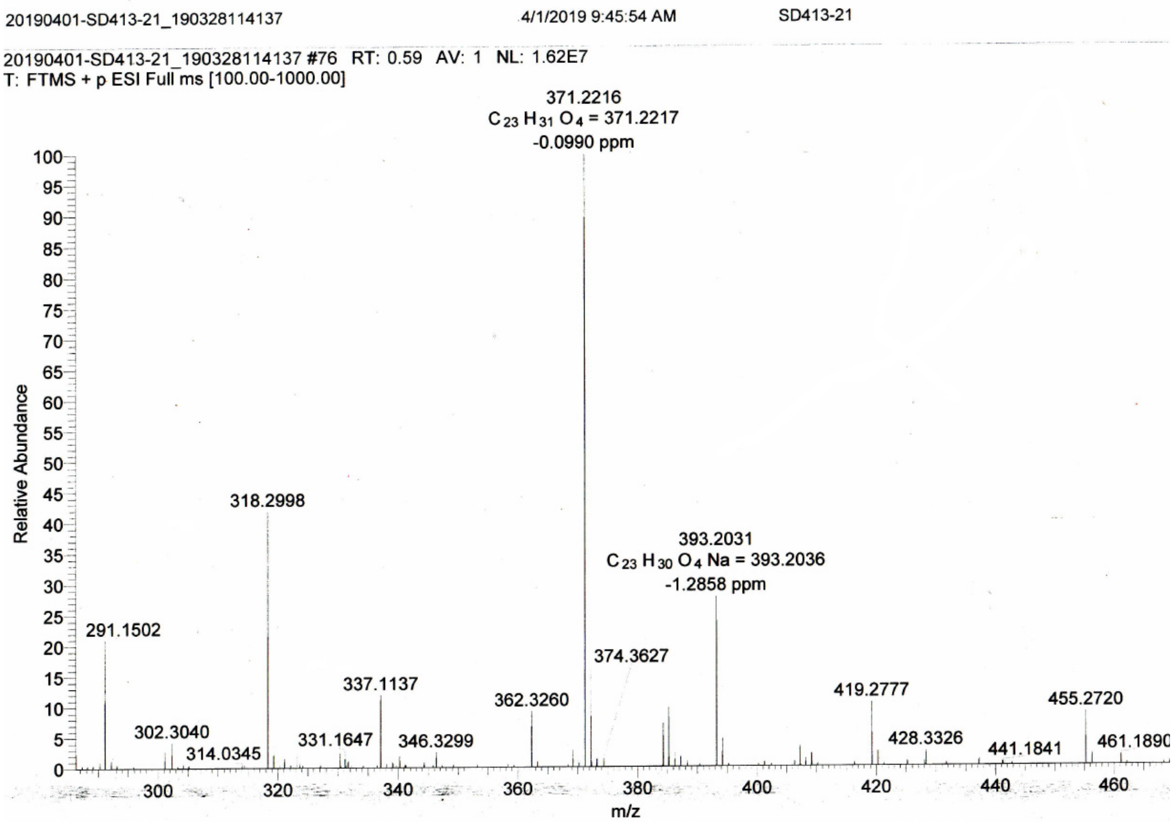

Figure S9. ^{1}H-NMR (500 MHz, DMSO-d_6) spectrum of compound **2**.

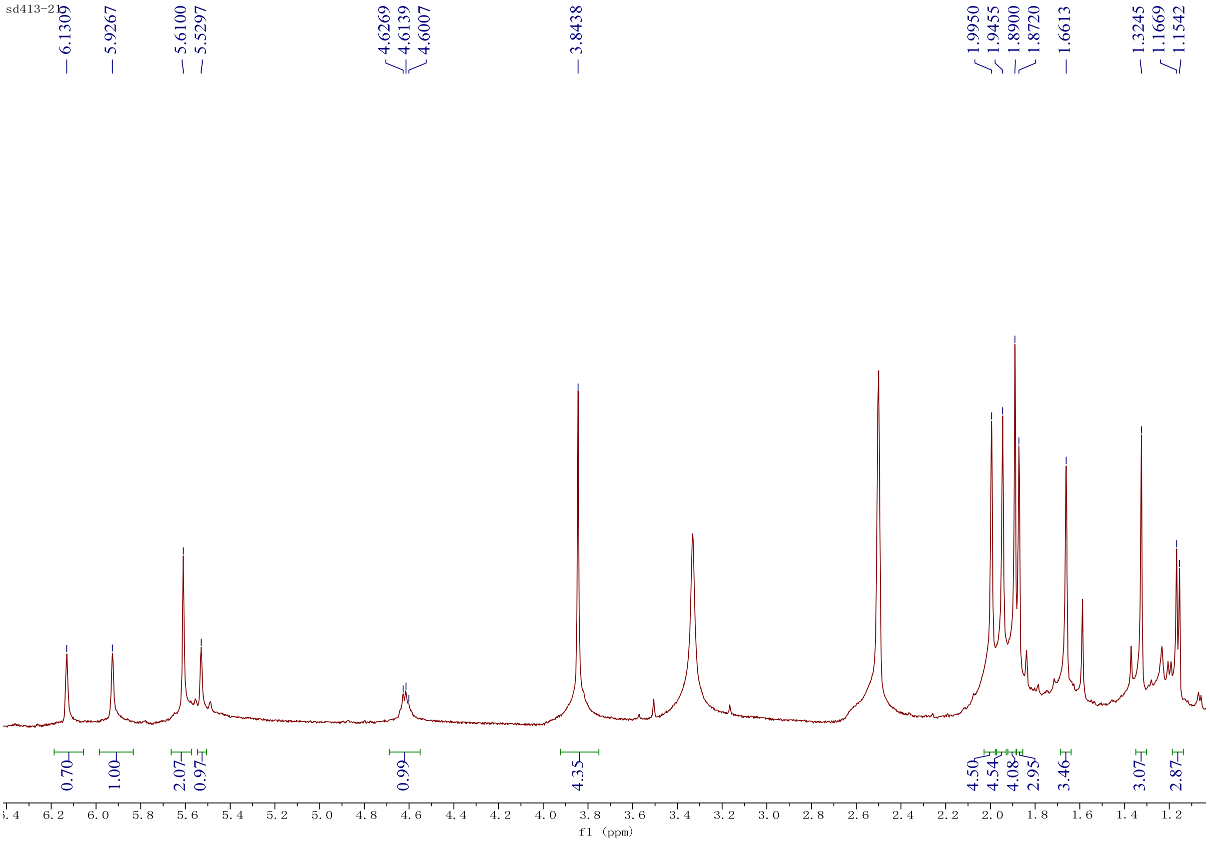

Figure S10. ^{13}C-NMR (125 MHz, DMSO-d_6) and DEPT spectra of compound **2**.

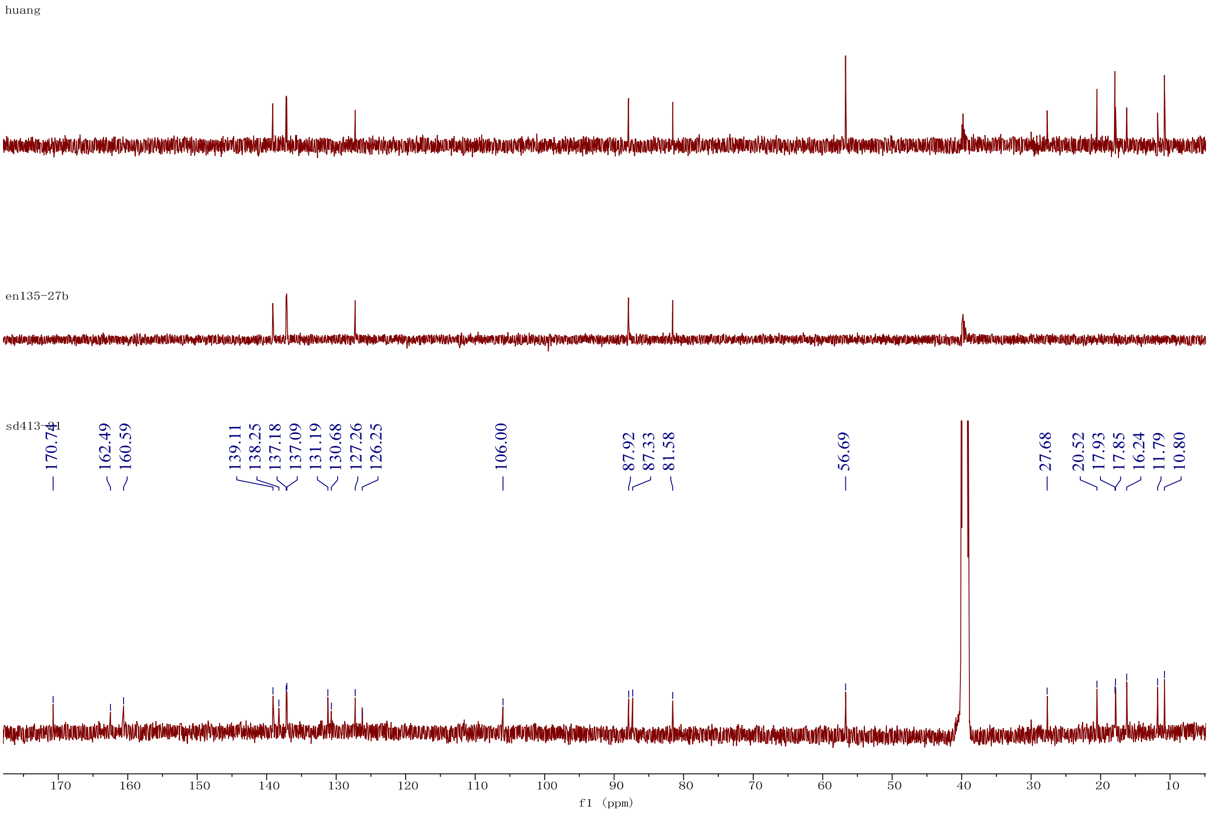

Figure S11. COSY spectrum of compound **2**.

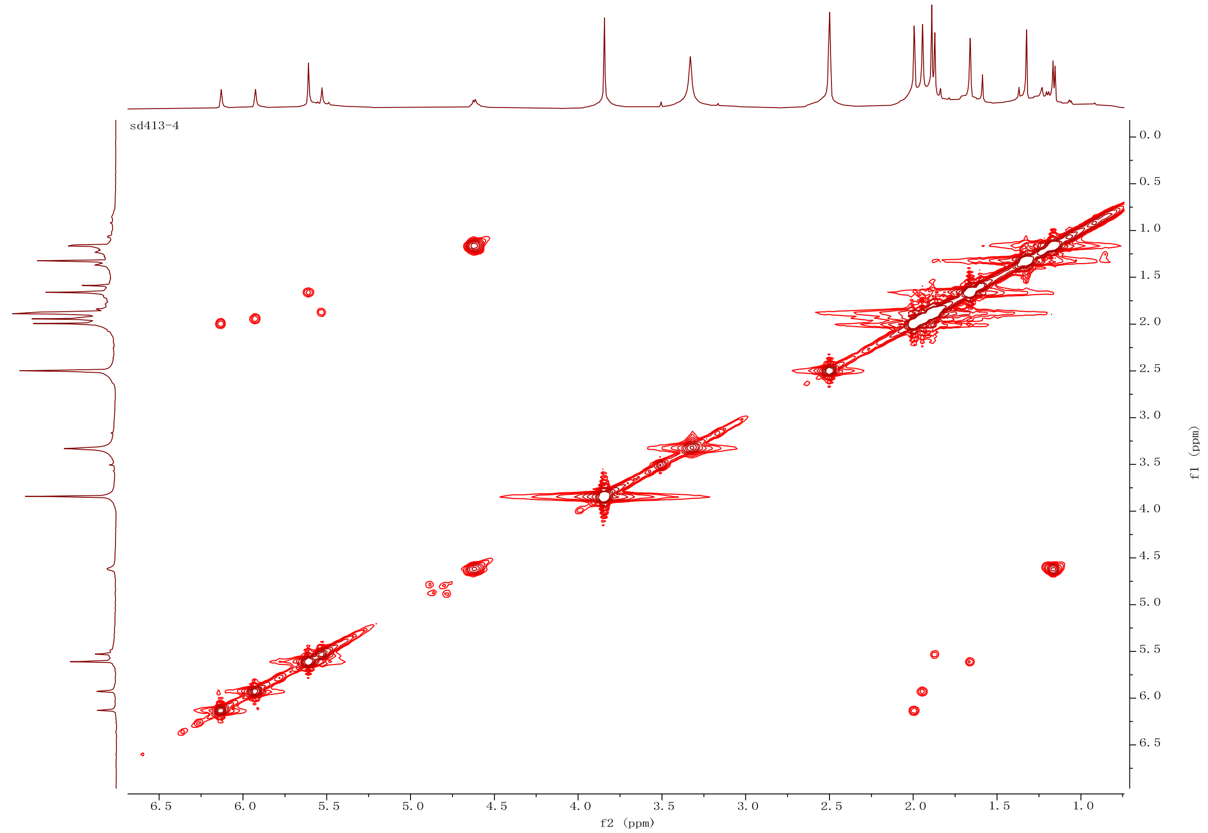

Figure S12. HSQC spectrum of compound **2**.

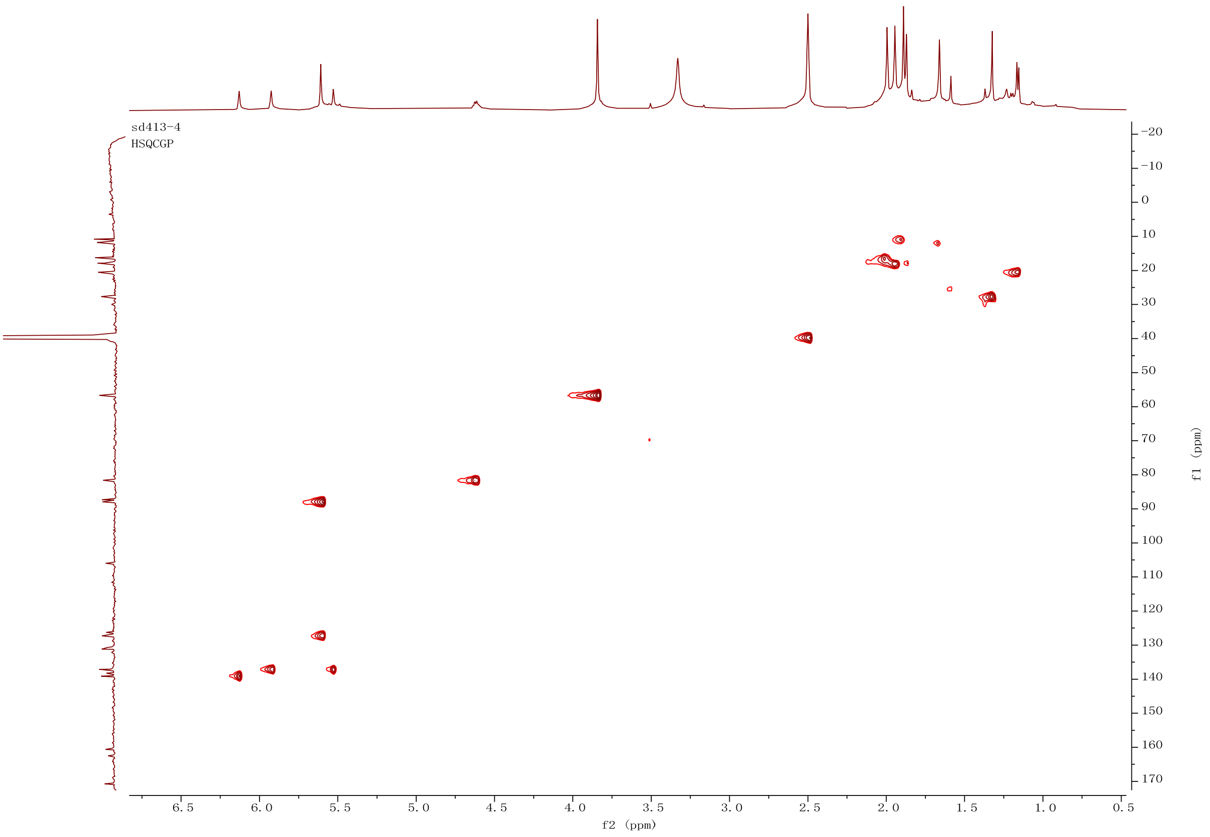

Figure S13. HMBC spectrum of compound **2**.

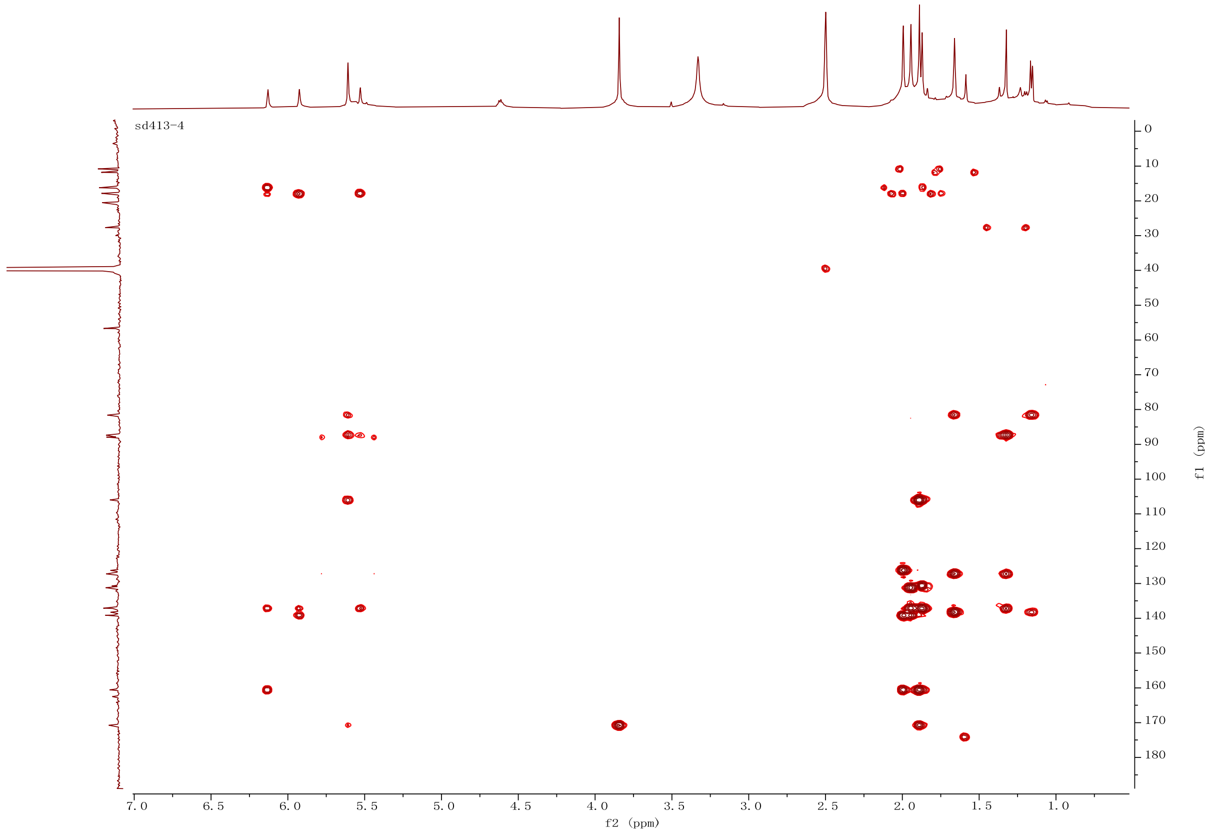

Figure S14. NOESY spectrum of compound **2**.

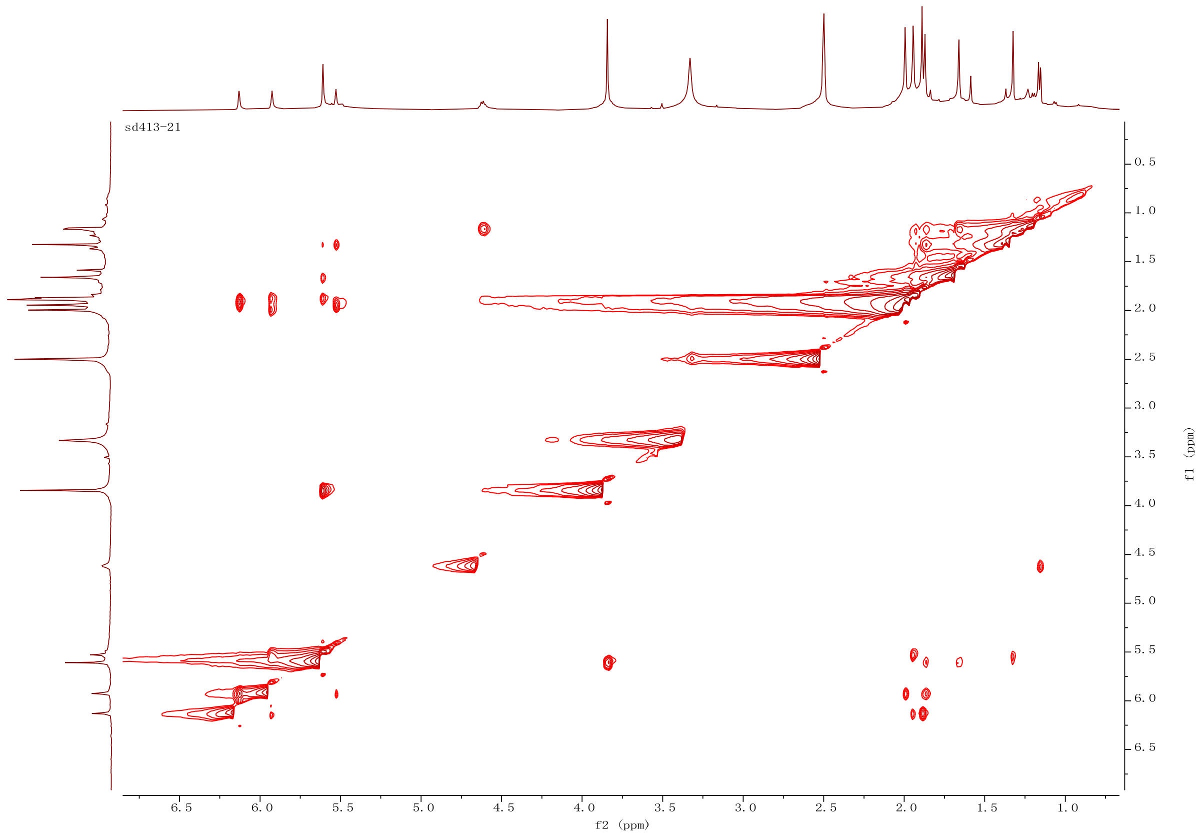

Figure S15. HRESIMS spectrum of compound **3**.

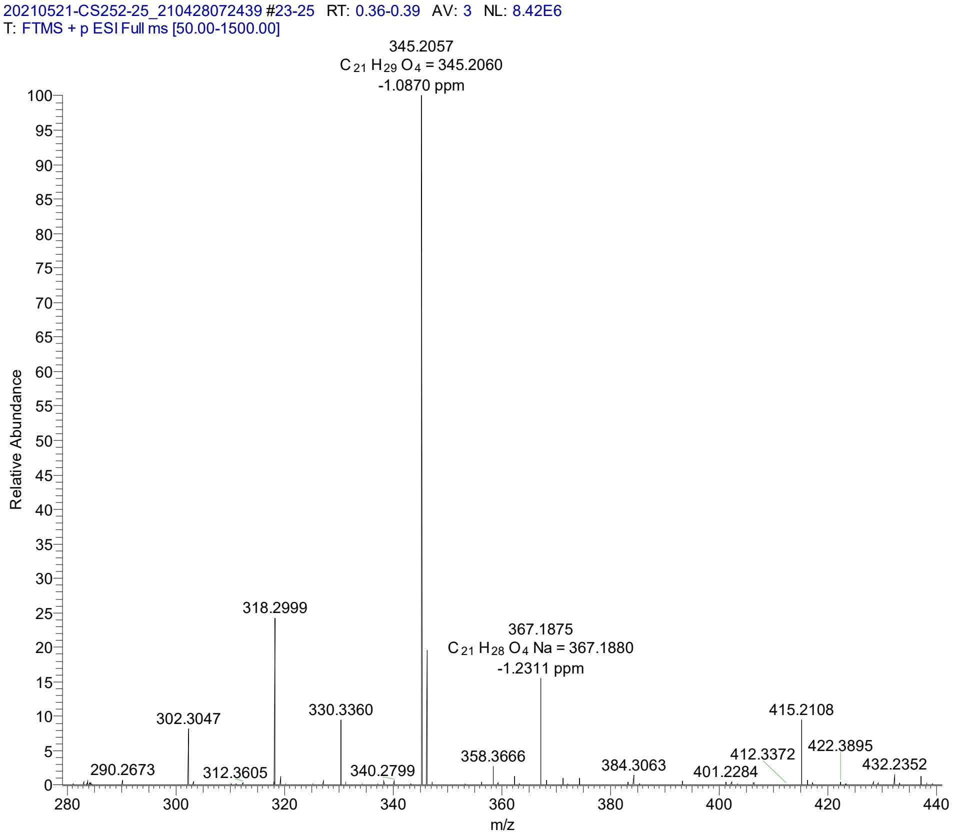

Figure S16. ^{1}H-NMR (500 MHz, DMSO-d_6) spectrum of compound **3**.

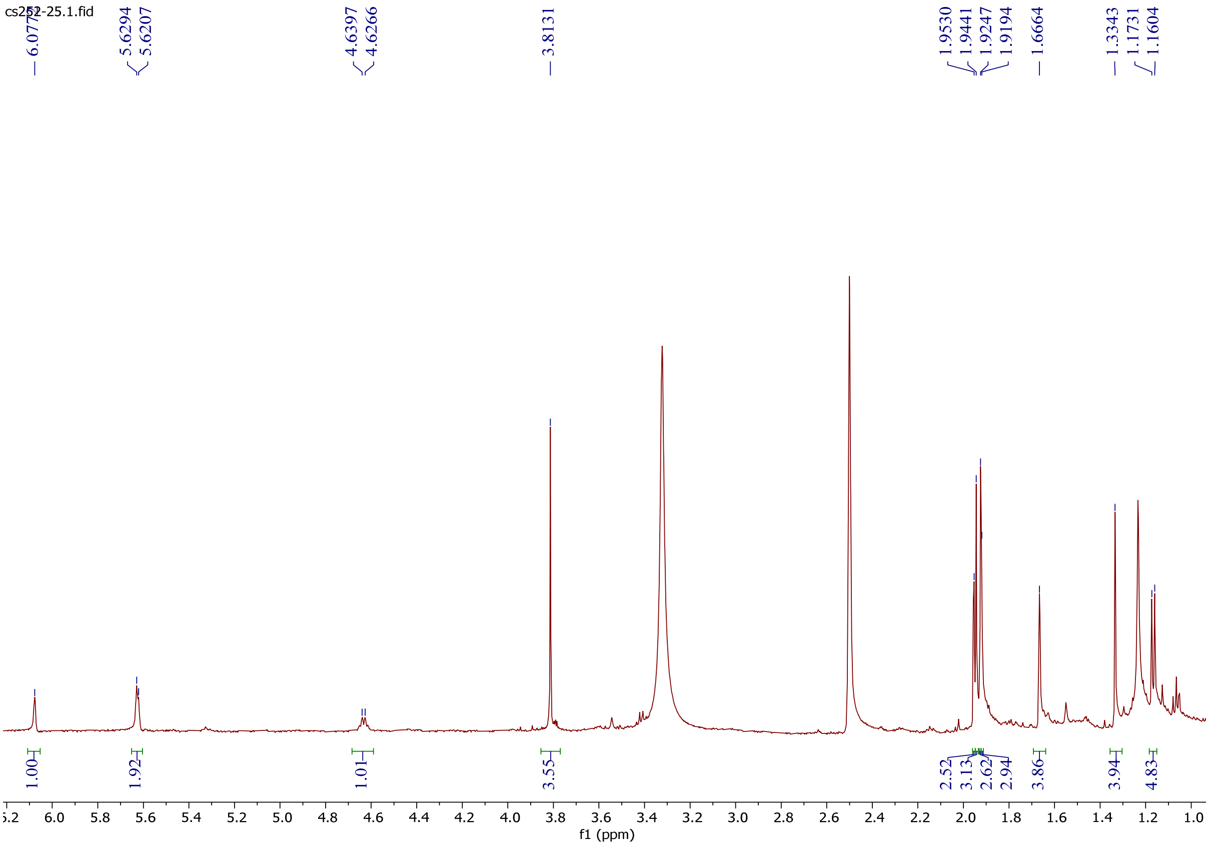

Figure S17. ^{13}C-NMR (125 MHz, DMSO-d_6) and DEPT spectra of compound **3**.

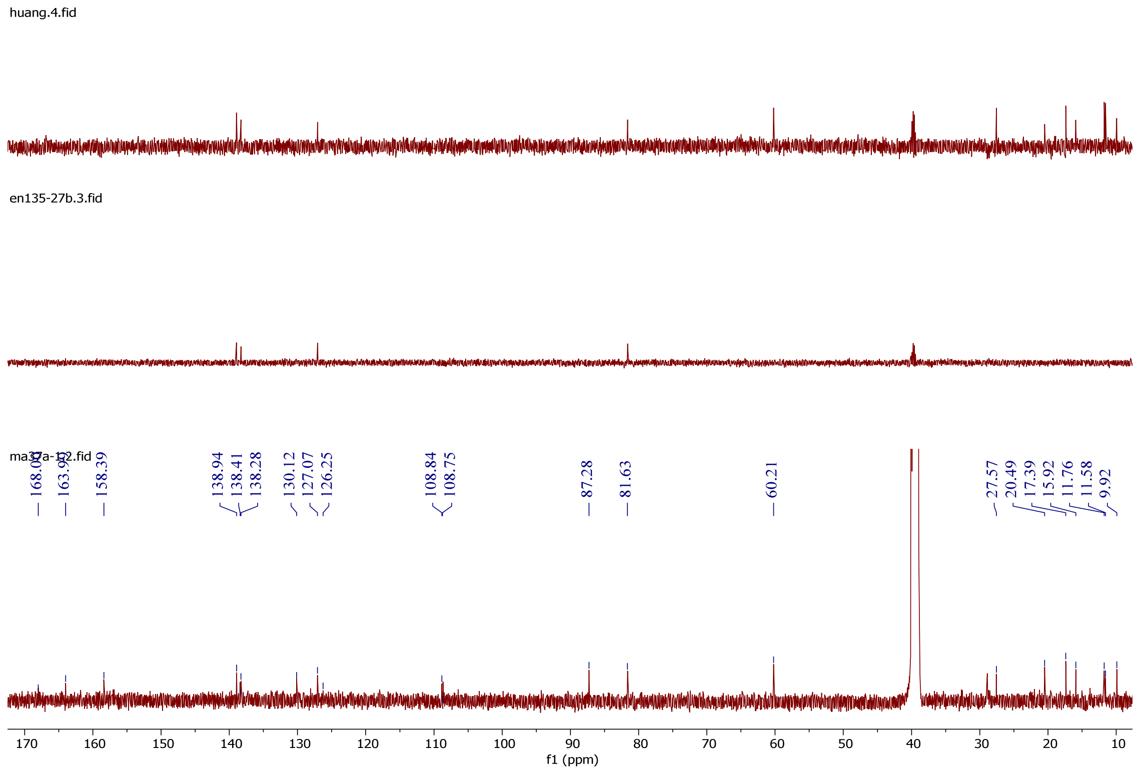

Figure S18. COSY spectrum of compound **3**.

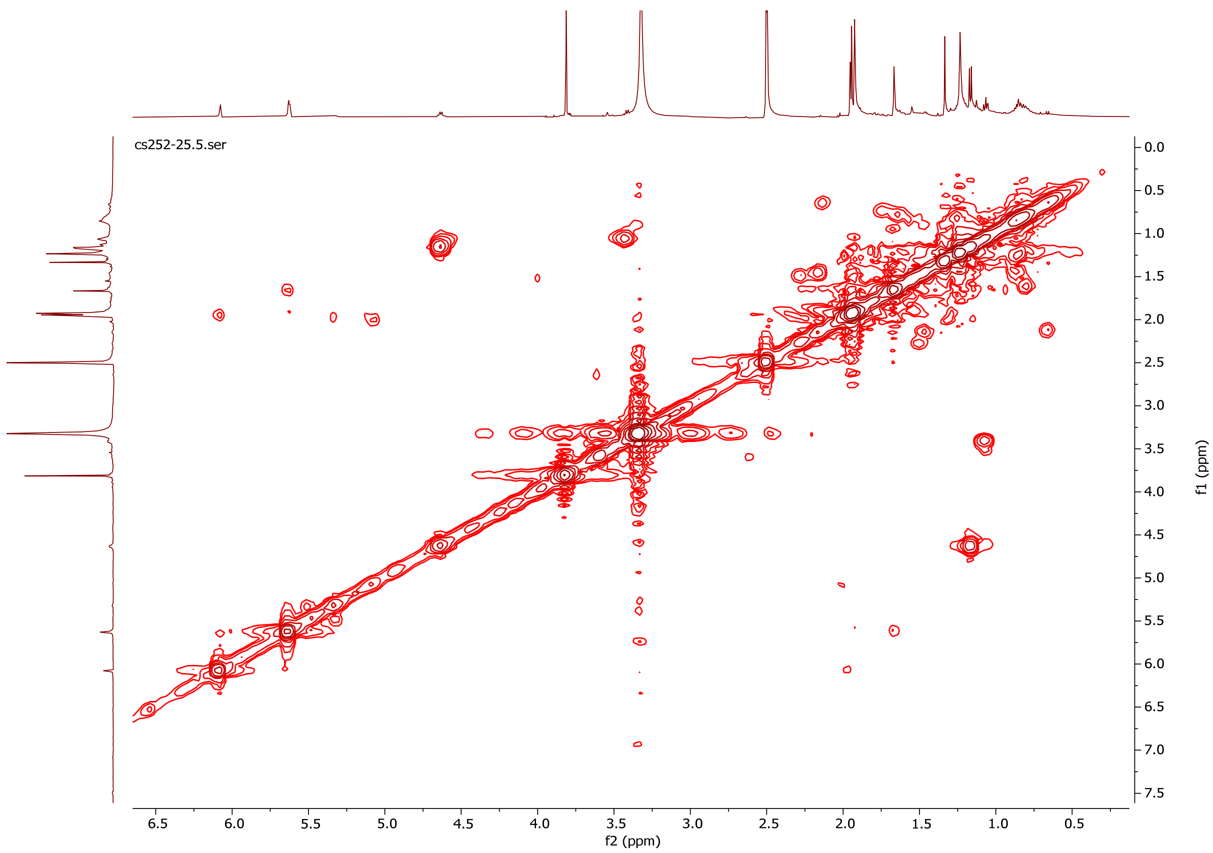

Figure S19. HSQC spectrum of compound **3**.

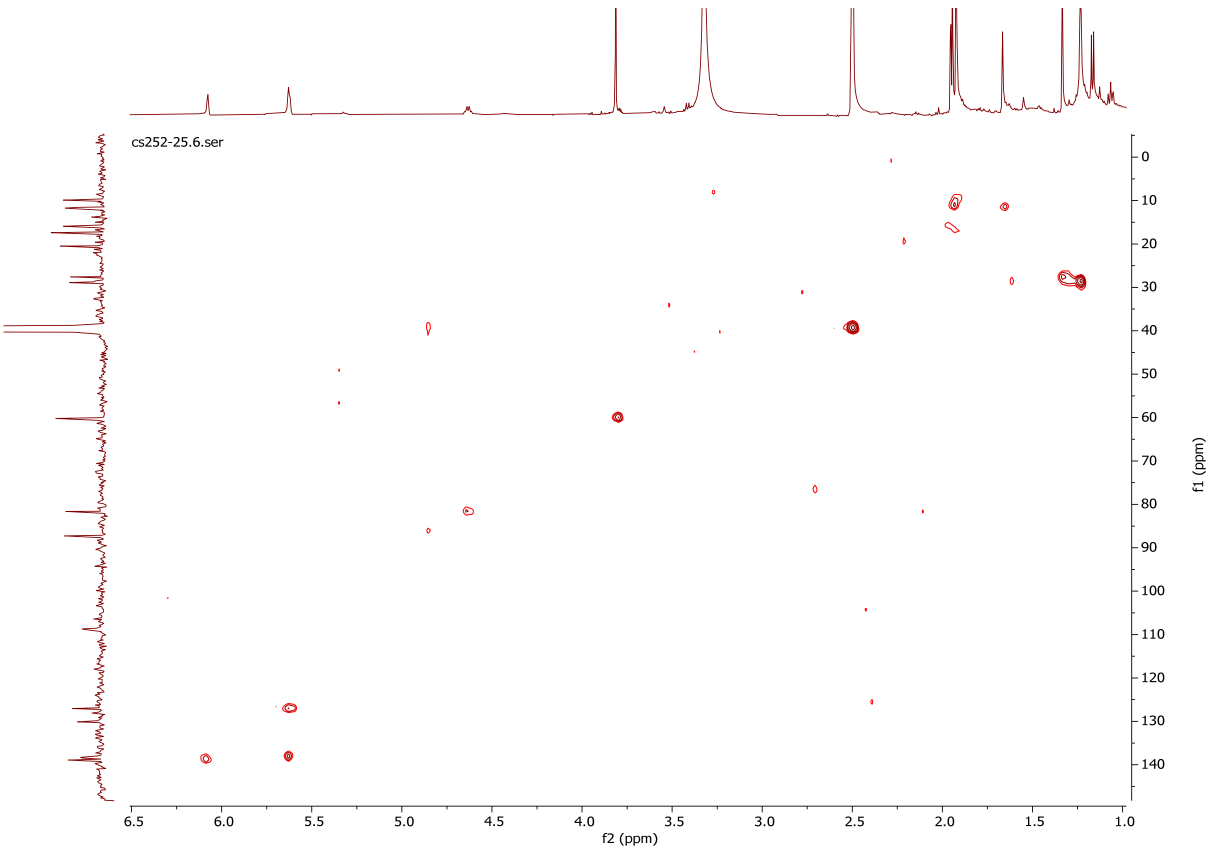

Figure S20. HMBC spectrum of compound **3**.

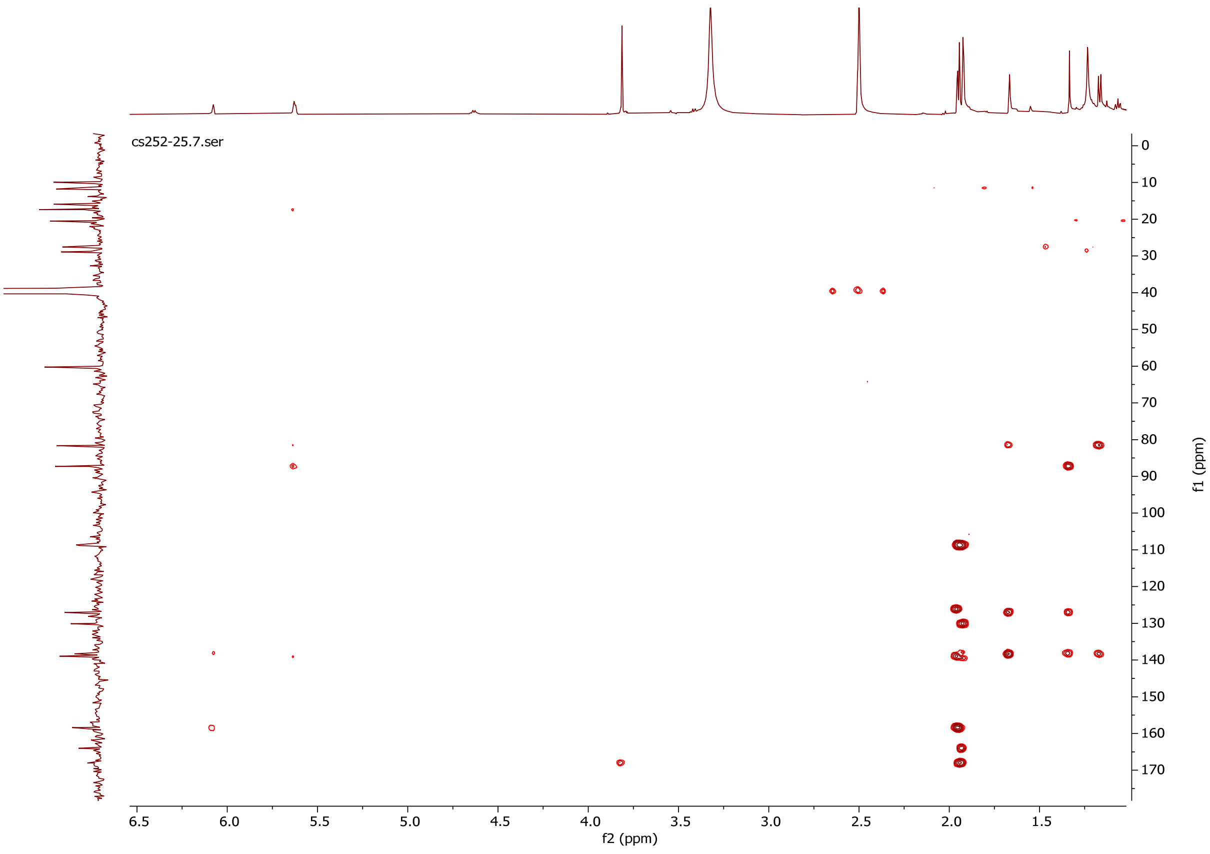

Figure S21. NOESY spectrum of compound **3**.

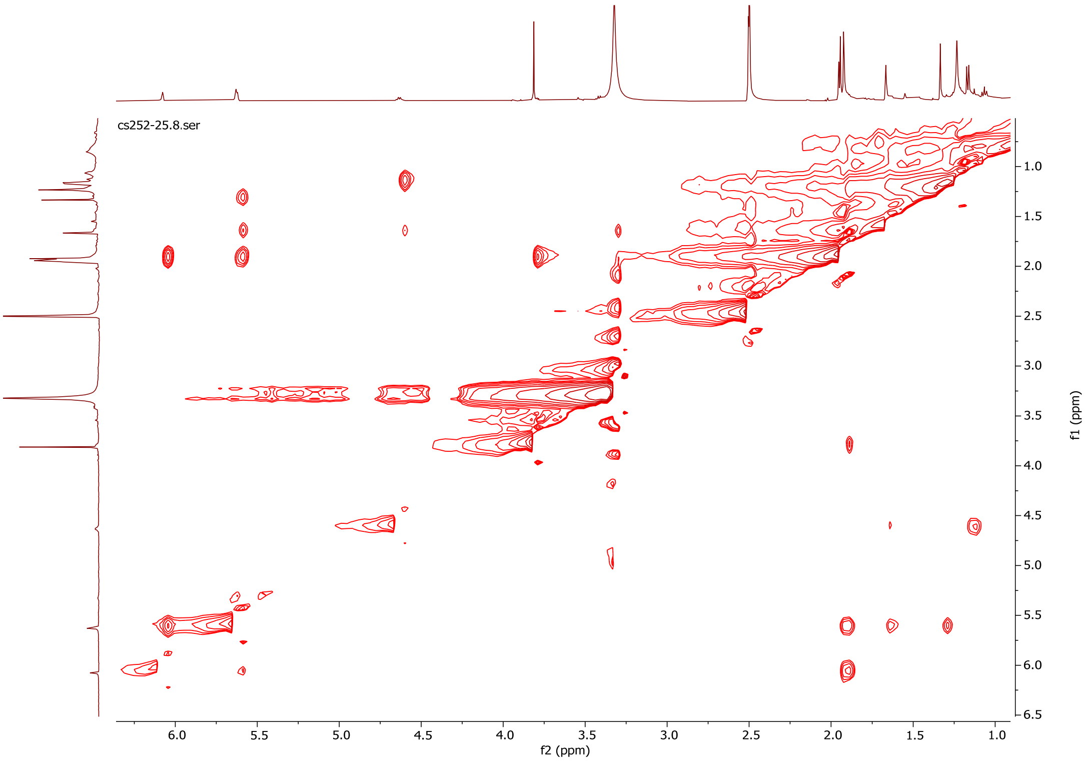

Figure S22. HRESIMS spectrum of compound **4**.

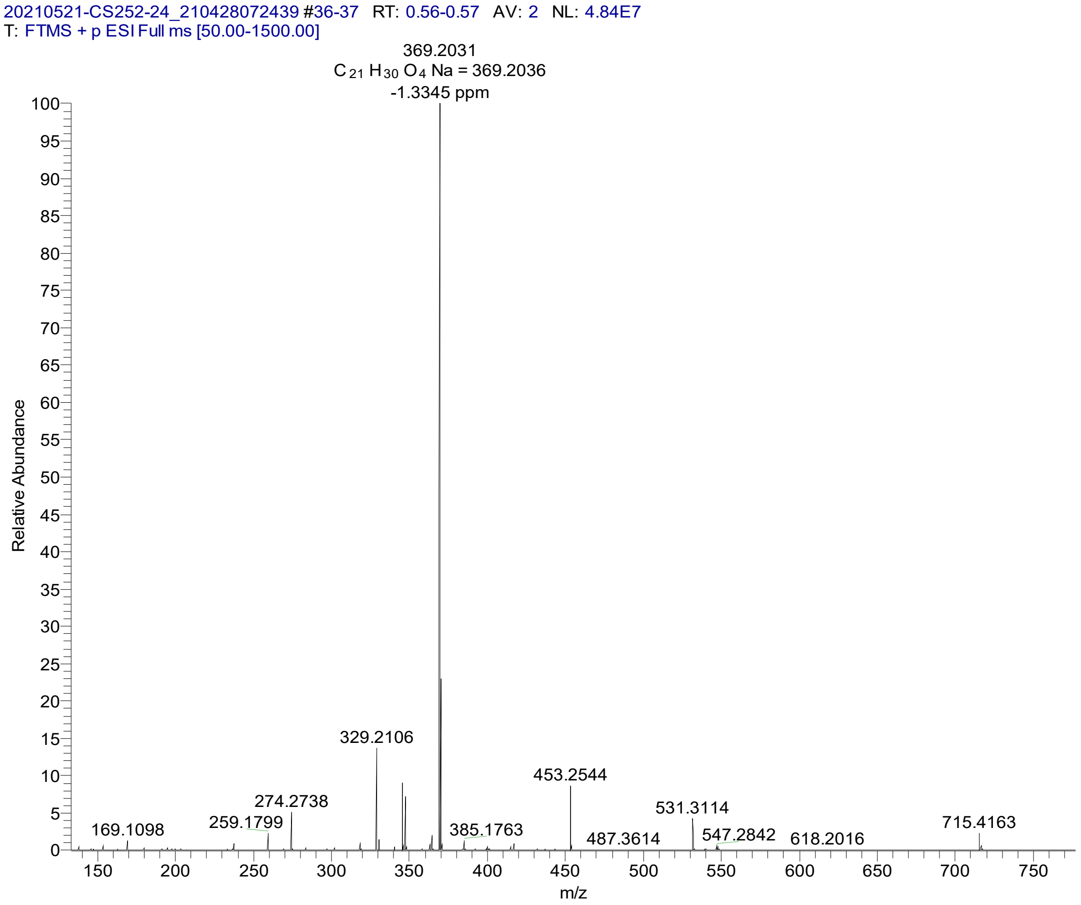

Figure S23. ^{1}H-NMR (500 MHz, DMSO-d_6) spectrum of compound **4**.

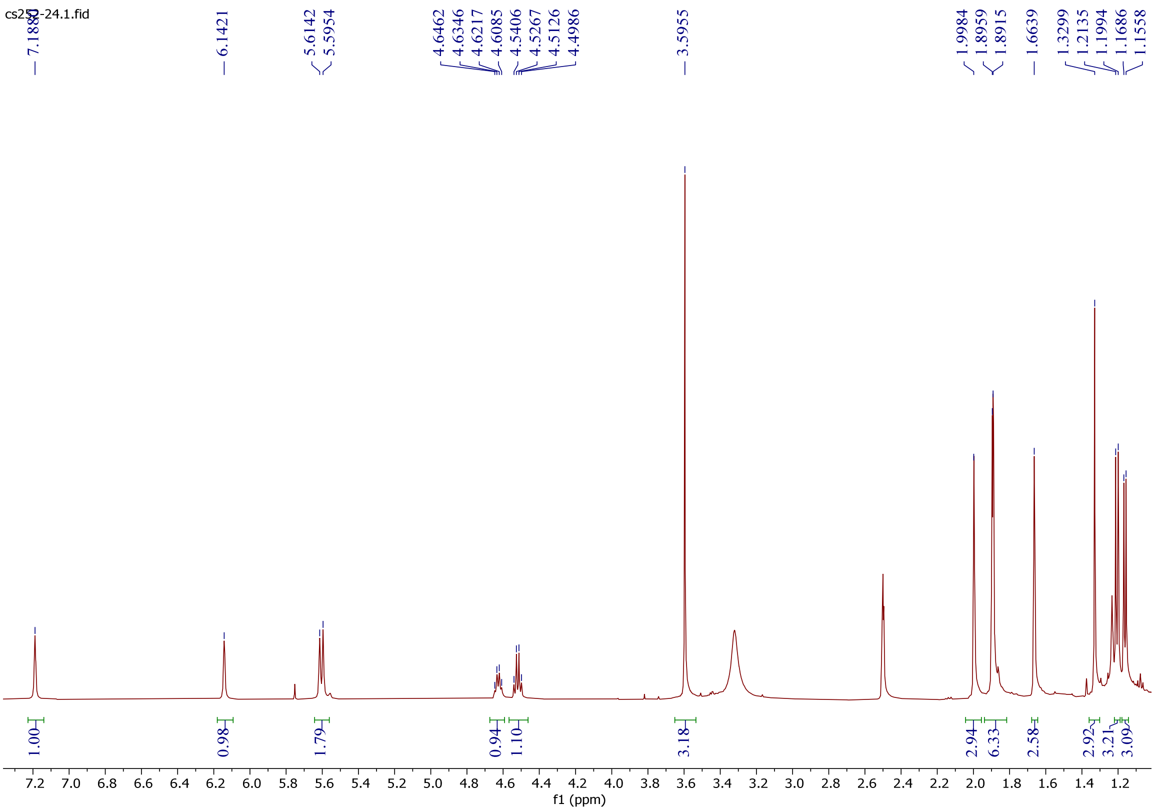

Figure S24. ^{13}C-NMR (125 MHz, DMSO-d_6) and DEPT spectra of compound **4**.

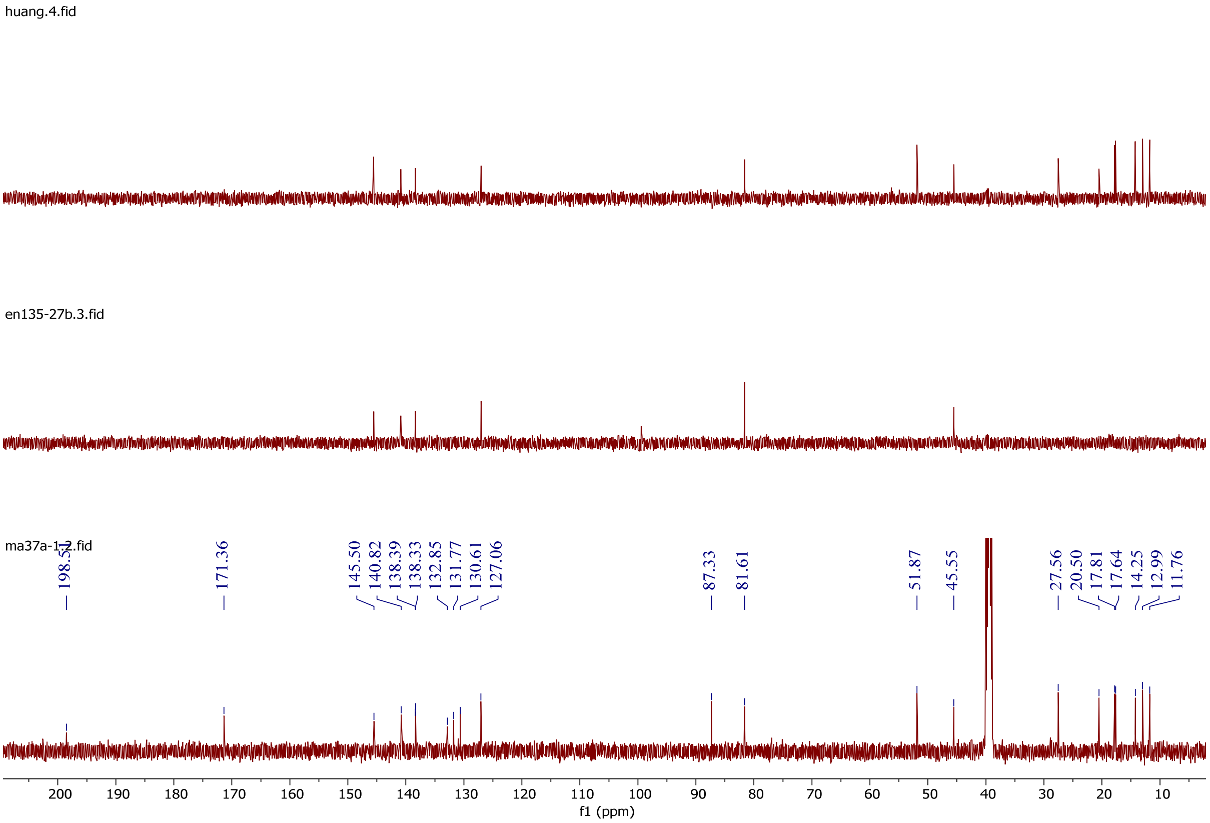

Figure S25. COSY spectrum of compound **4**.

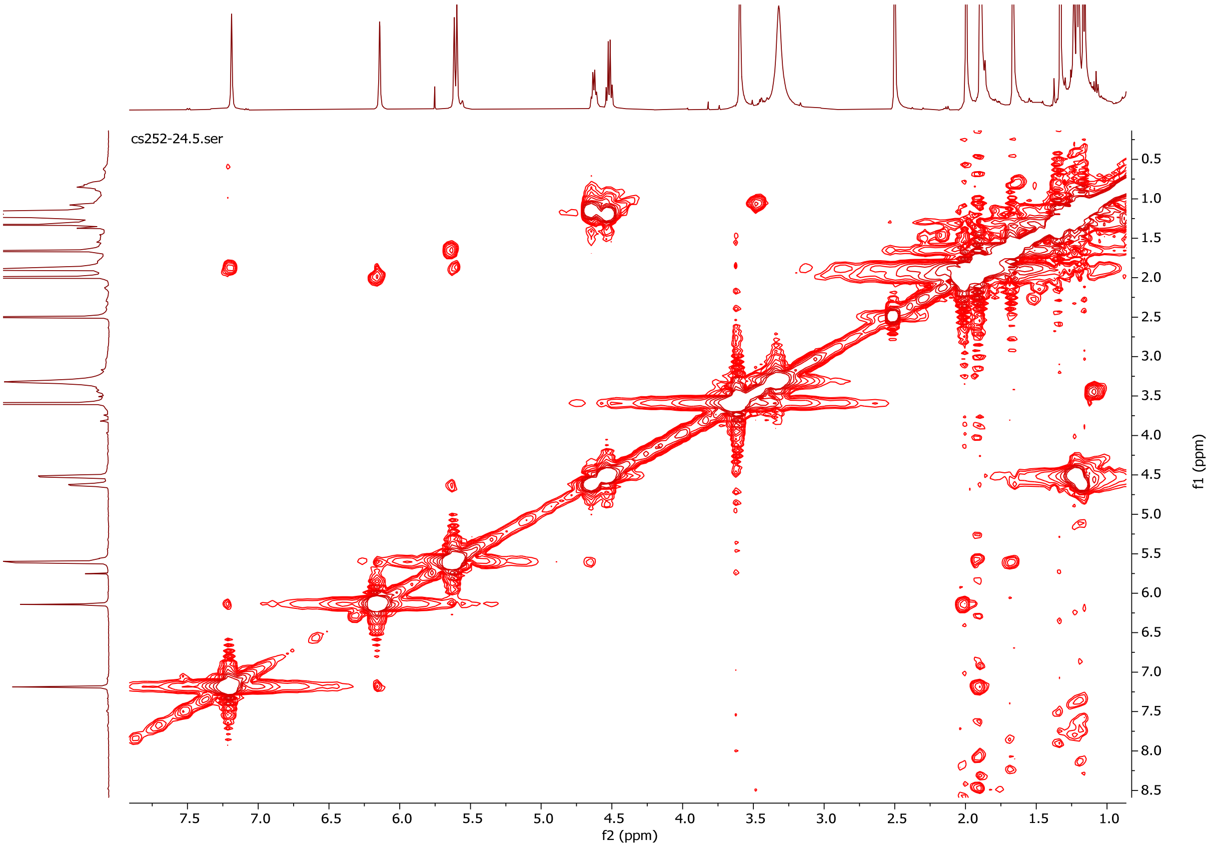

Figure S26. HSQC spectrum of compound **4**.

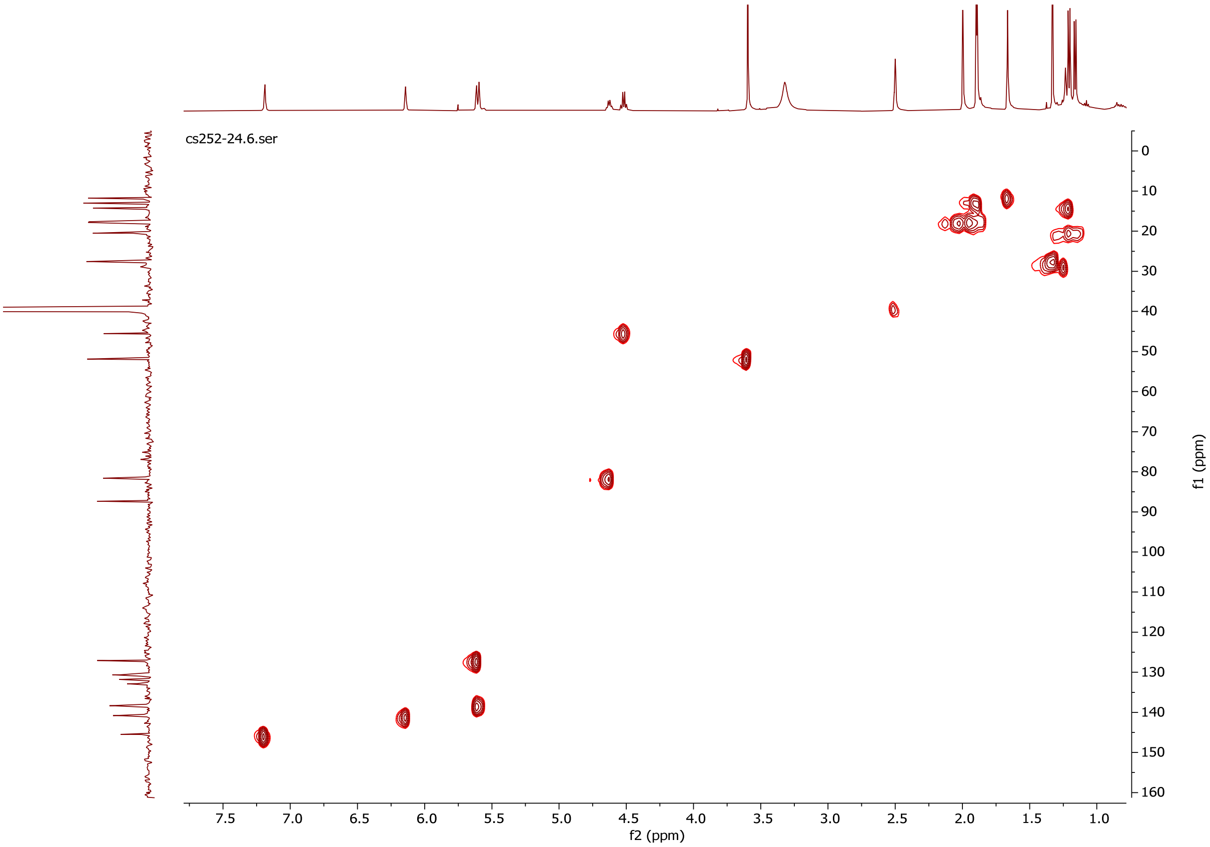

Figure S27. HMBC spectrum of compound **4**.

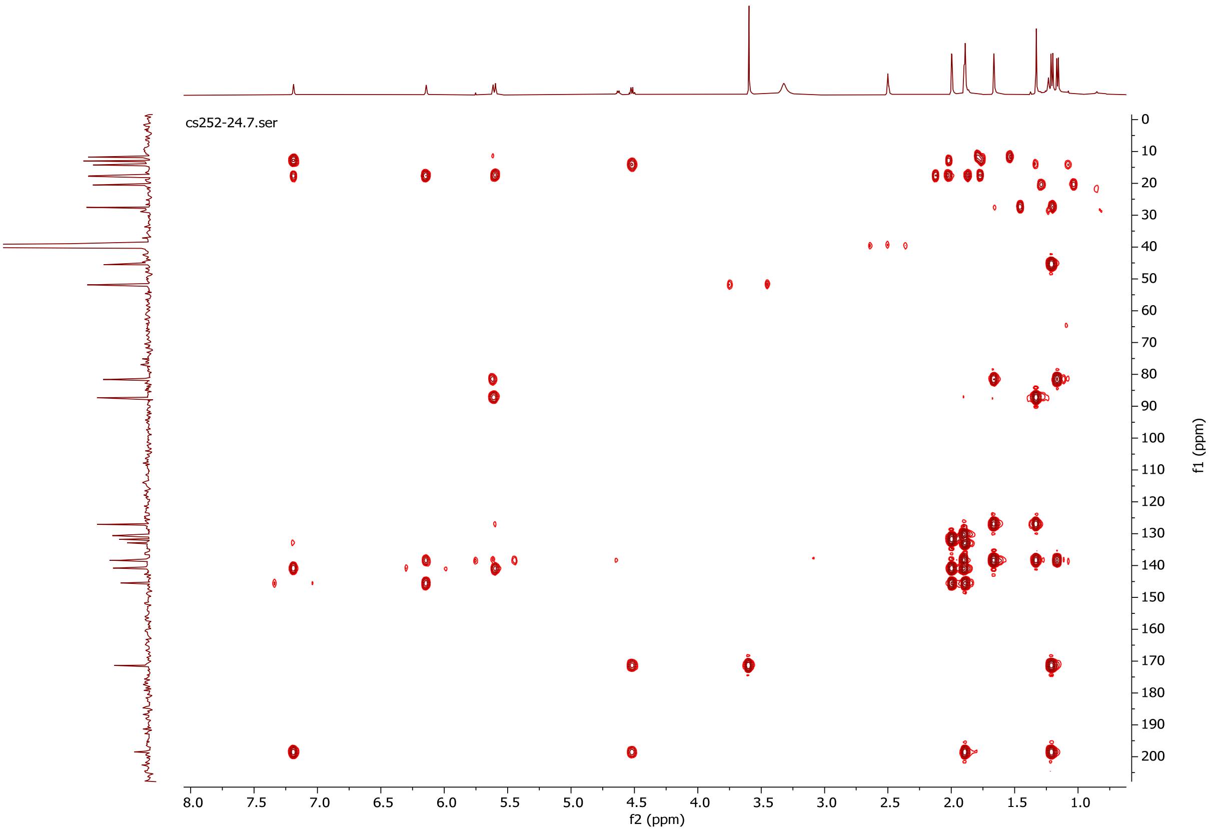

Figure S28. NOESY spectrum of compound **4**.

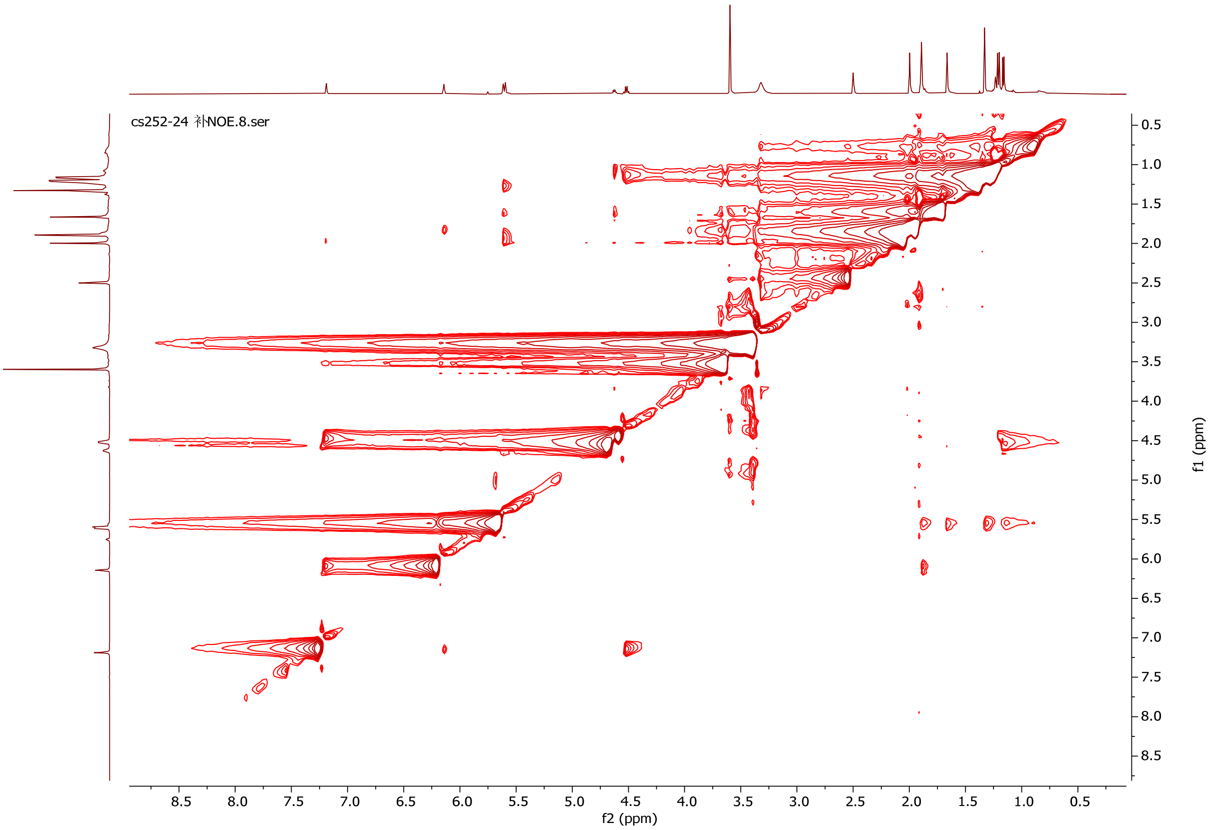

Figure S29. HRESIMS spectrum of compound **5**.

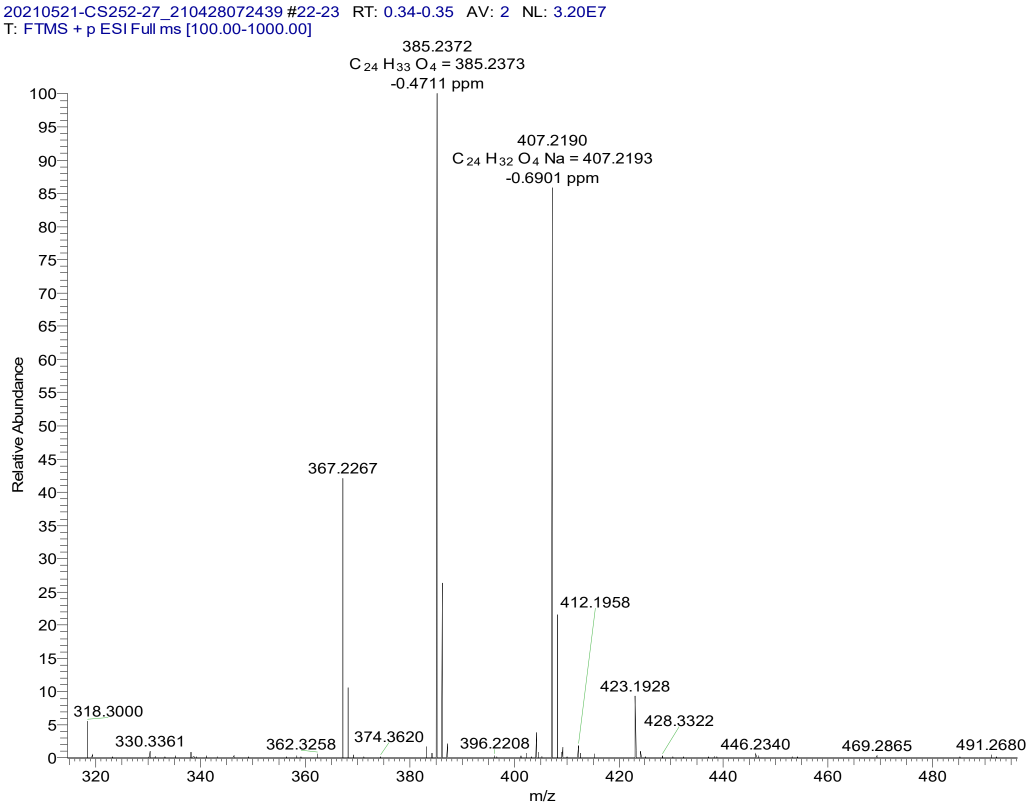

Figure S30. ^{1}H-NMR (500 MHz, DMSO-d_6) spectrum of compound **5**.

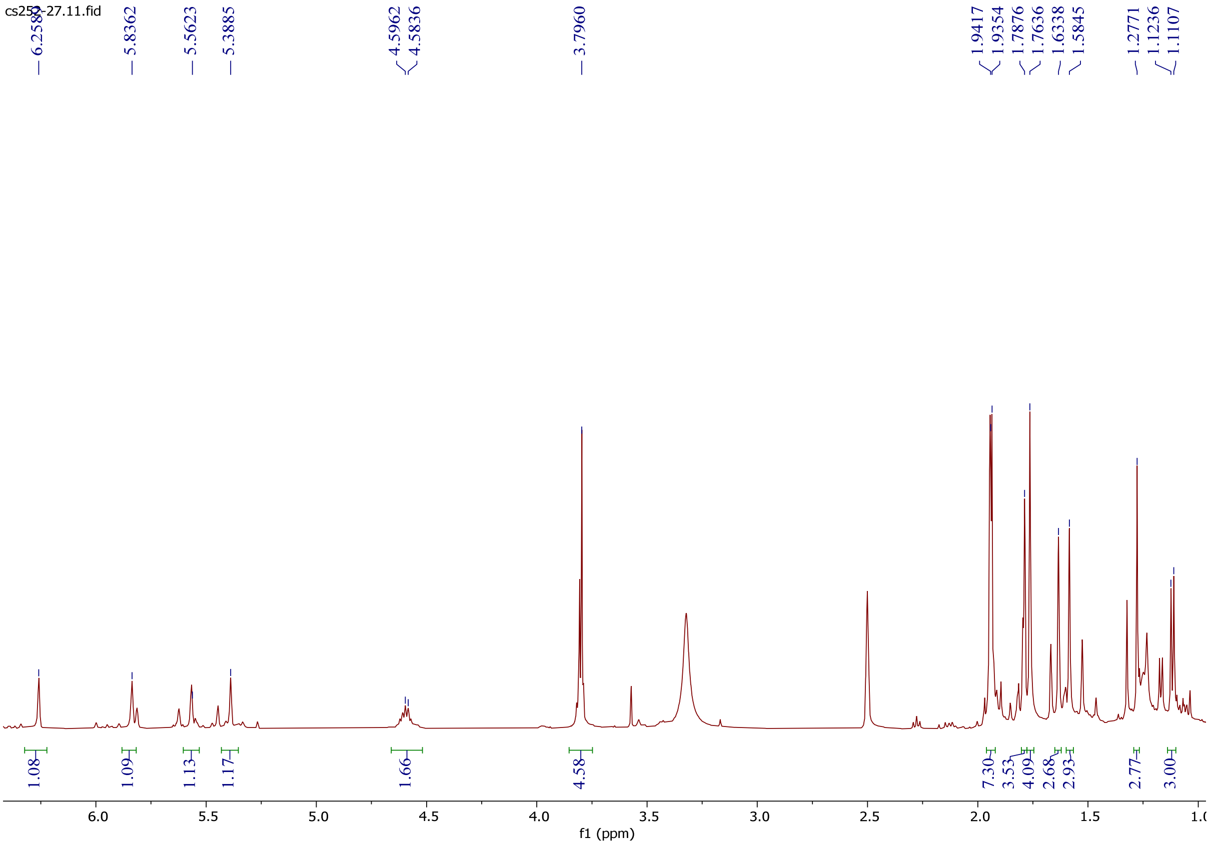

Figure S31. ^{13}C-NMR (125 MHz, DMSO-d_6) and DEPT spectra of compound **5**.

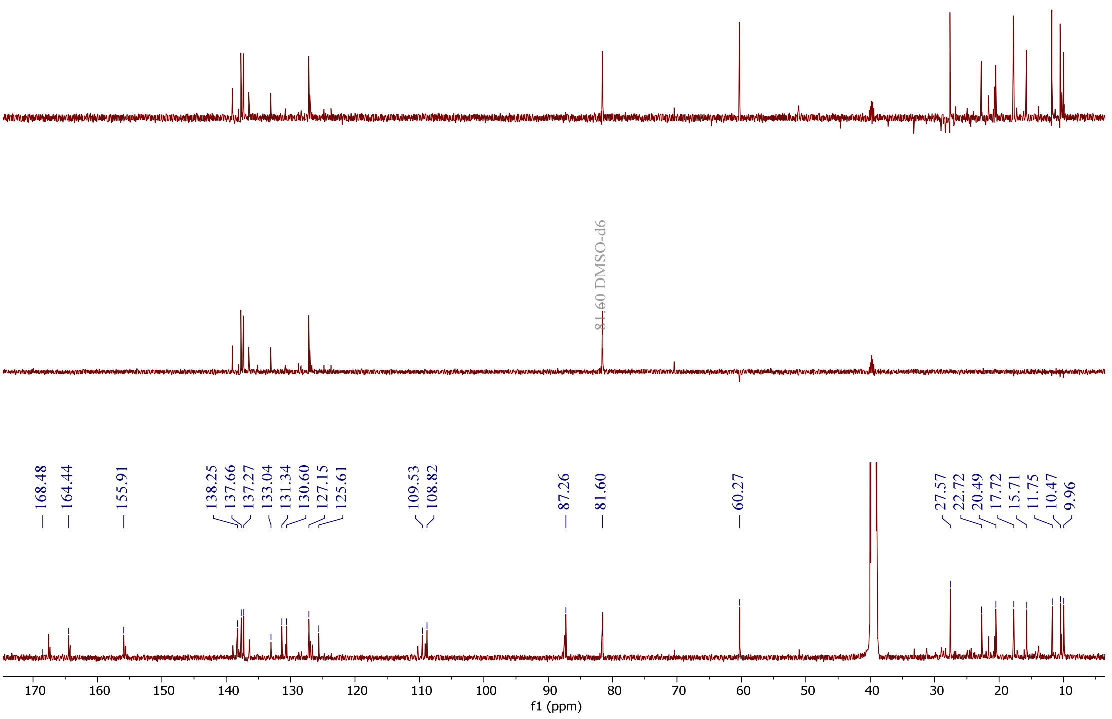

Figure S32. COSY spectrum of compound **5**.

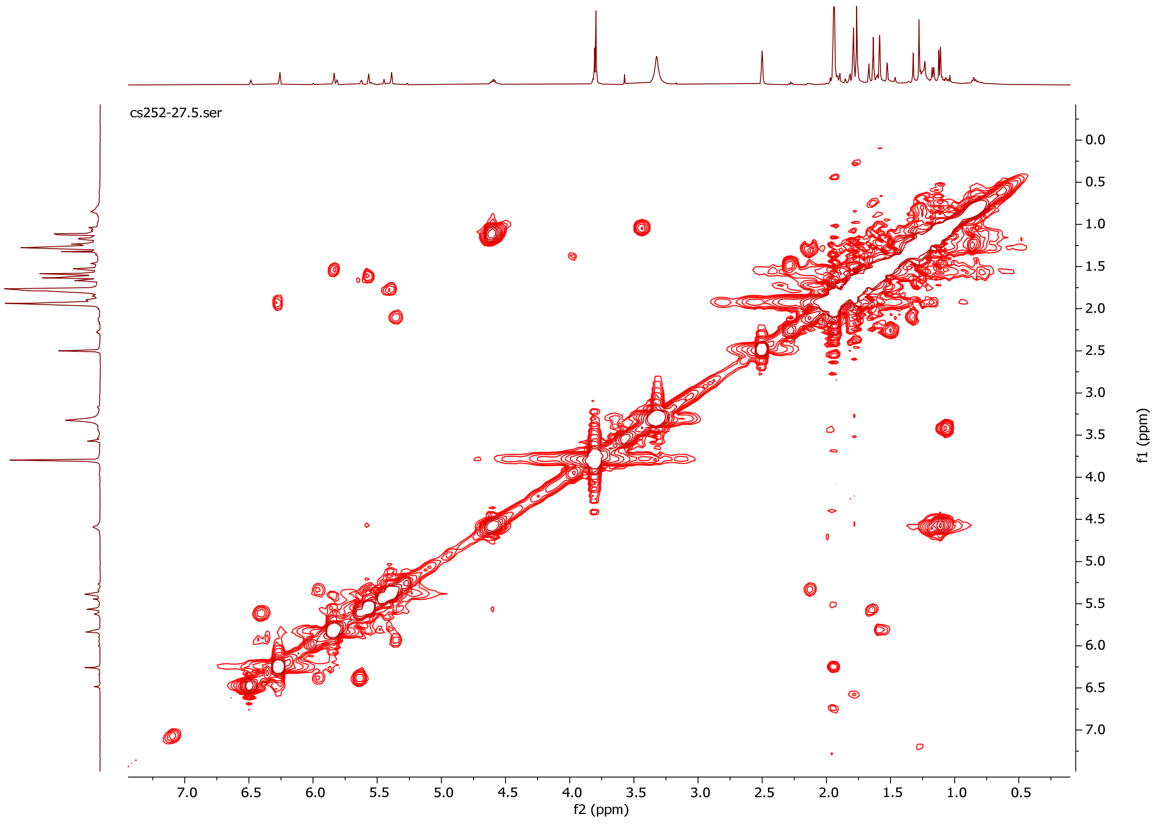

Figure S33. HSQC spectrum of compound **5**.

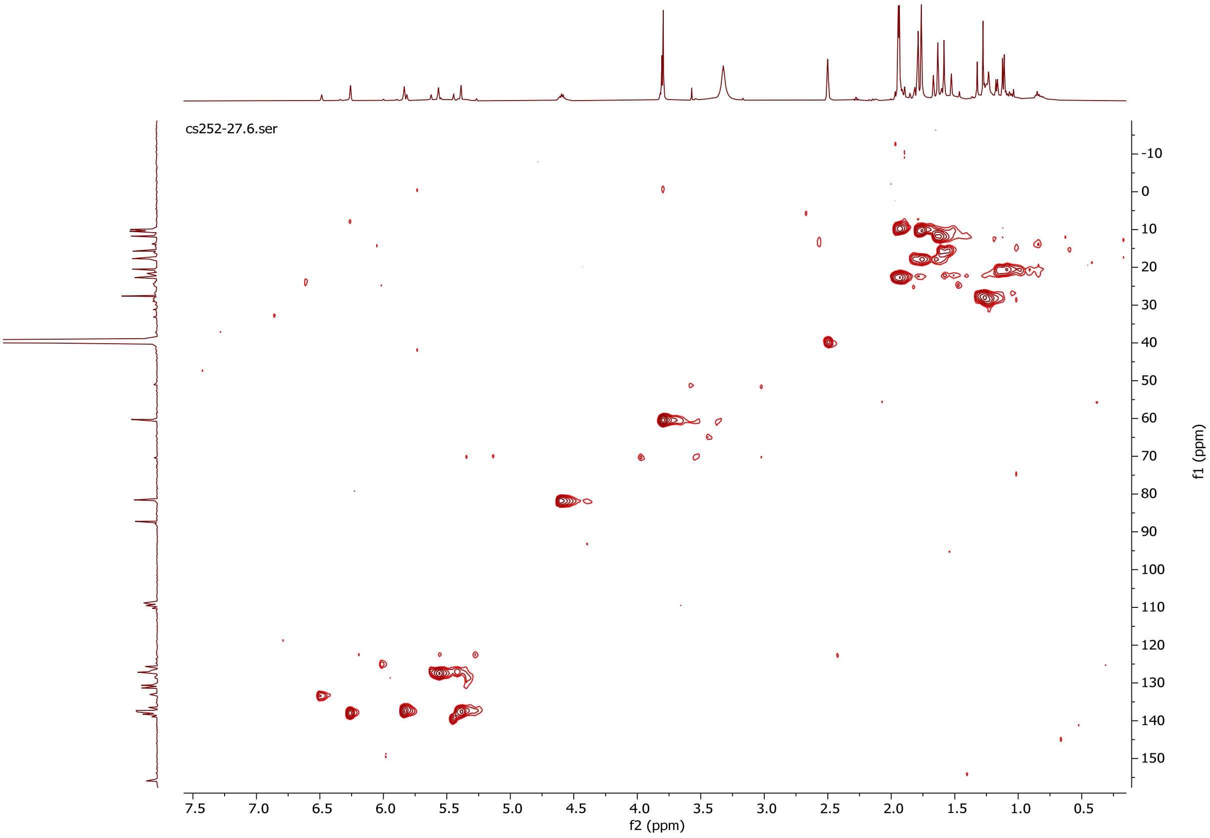

Figure S34. HMBC spectrum of compound **5**.

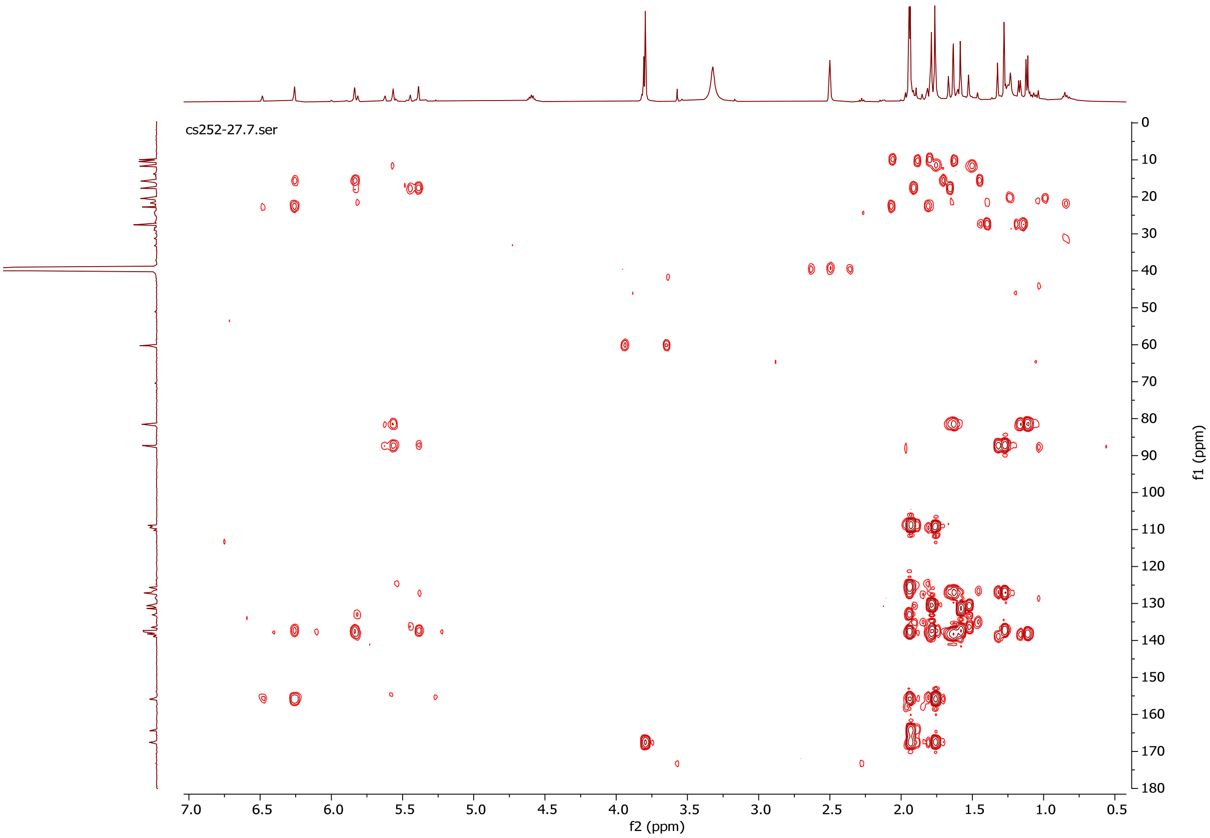

Figure S35. HRESIMS spectrum of compound **6**.

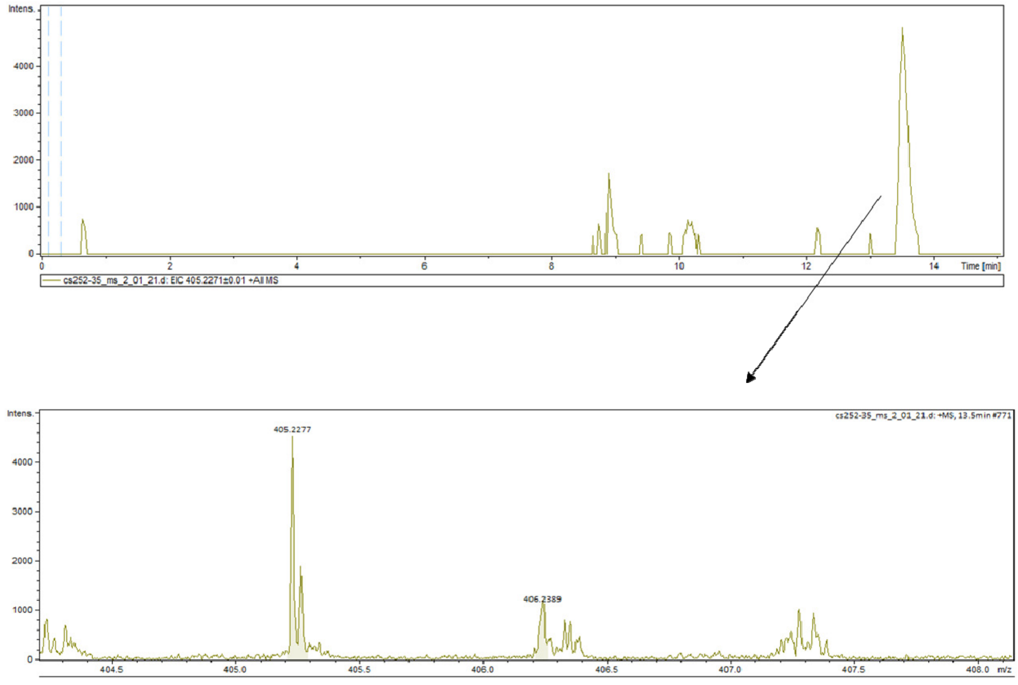

Meas. m/z	#	Ion Formula	Score	m/z	err [mDa]	err [ppm]	mSigma	rdb	e?Conf	N-Rule	Adduct	Sum Formula
405.227745	1	C23H33O6	100.00	405.227165	-0.6	-1.4	40.0	7.5	even	ok	M+H	C23H32O6

Figure S36. ^{1}H-NMR (500 MHz, DMSO-d_6) spectrum of compound **6**.

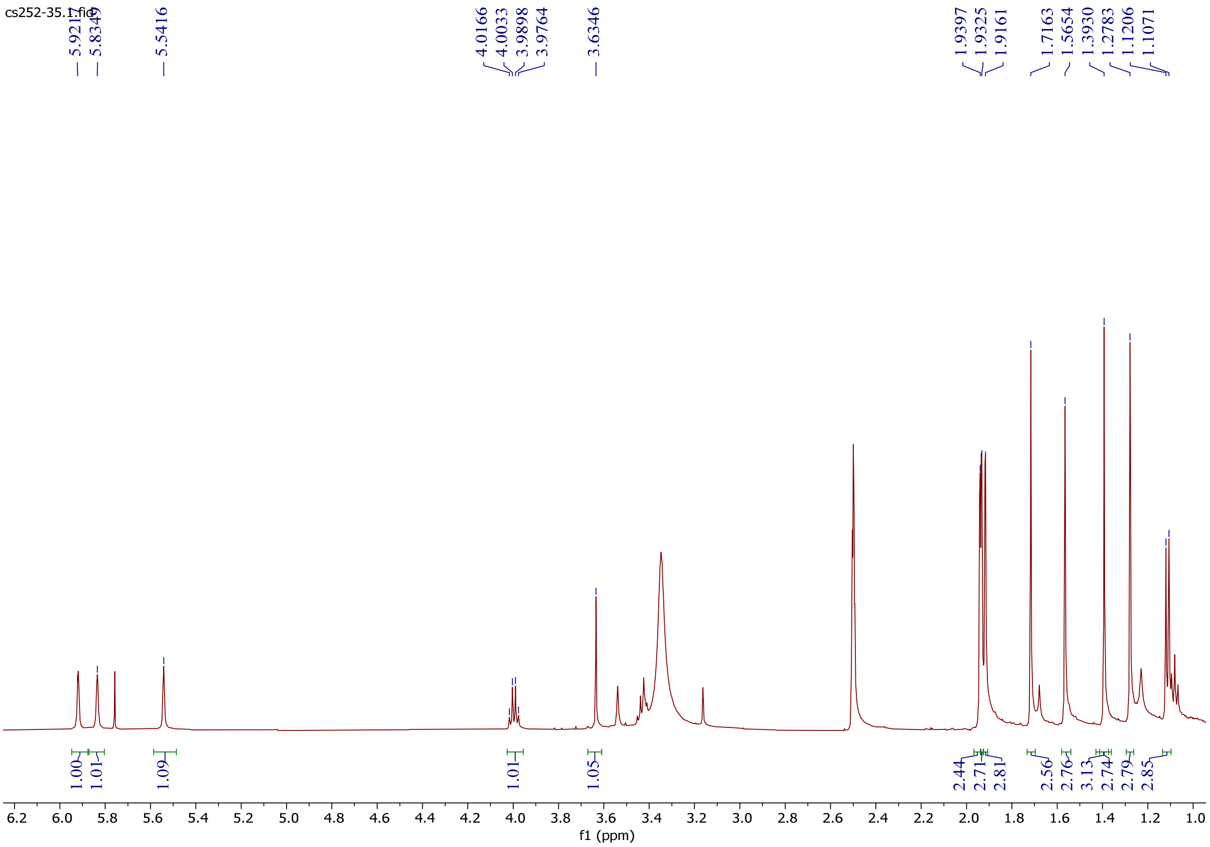

Figure S37. ^{13}C-NMR (125 MHz, DMSO-d_6) and DEPT spectra of compound **6**.

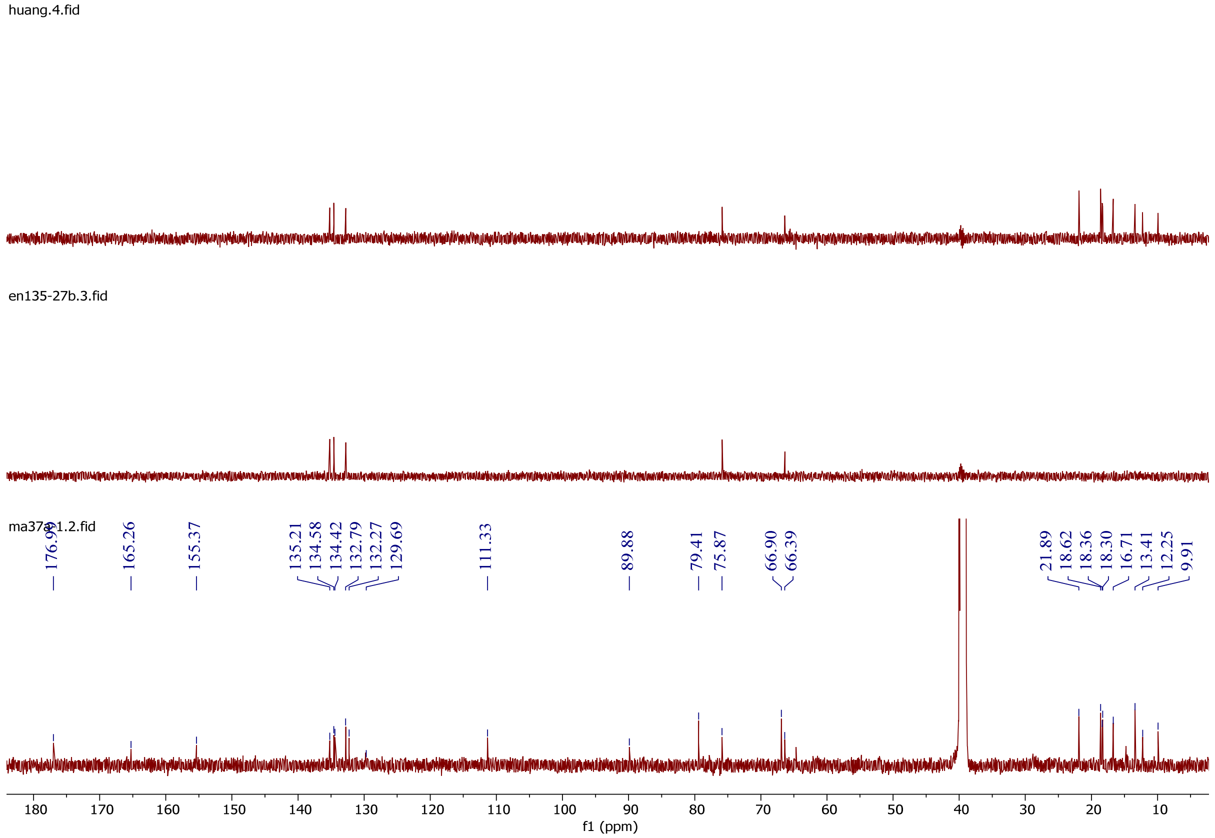

Figure S38. COSY spectrum of compound **6**.

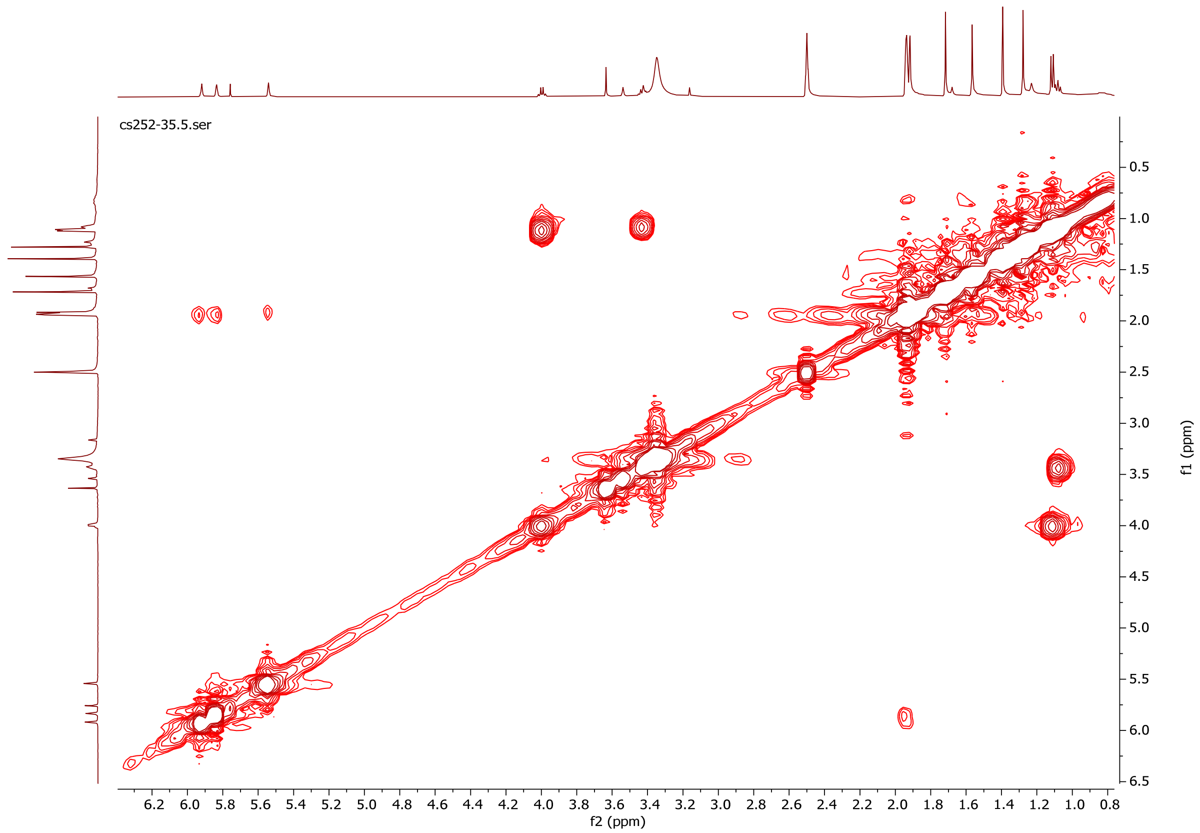

Figure S39. HSQC spectrum of compound **6**.

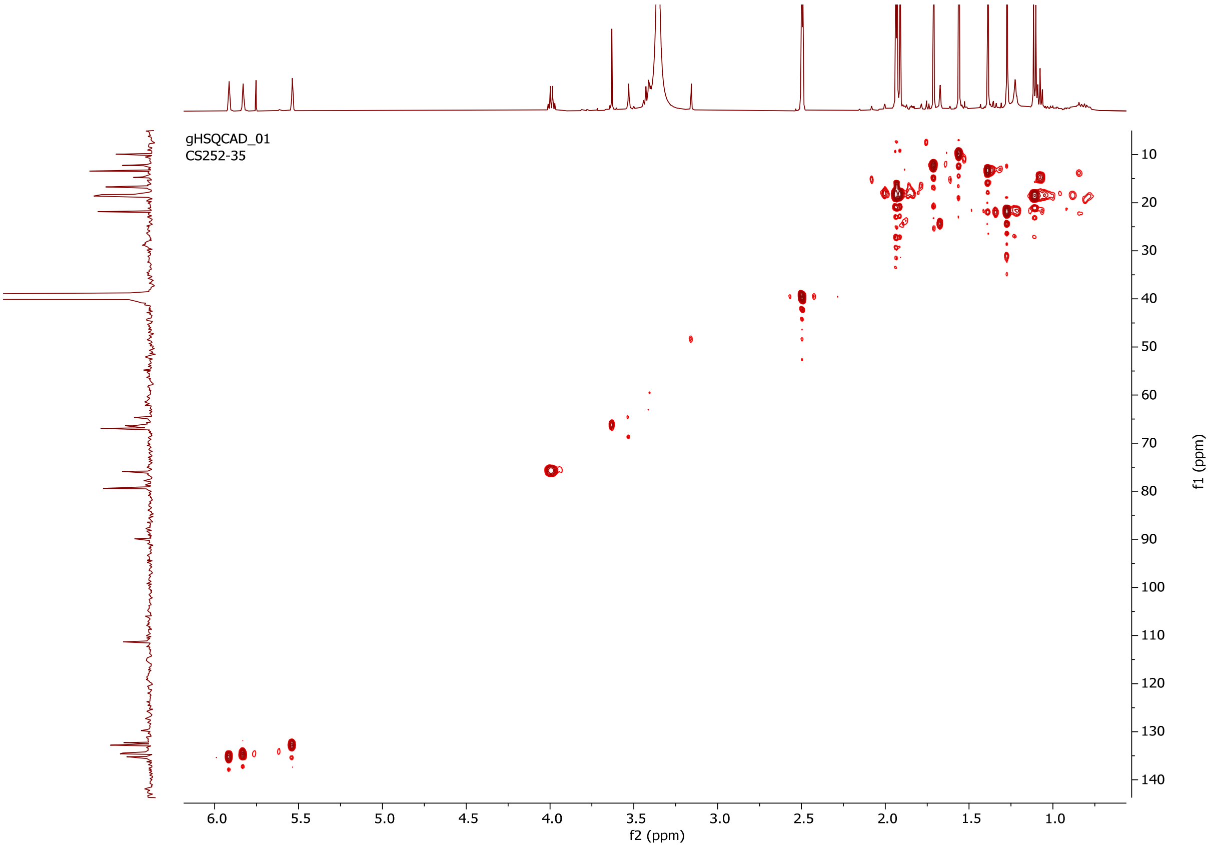

Figure S40. HMBC spectrum of compound **6**.

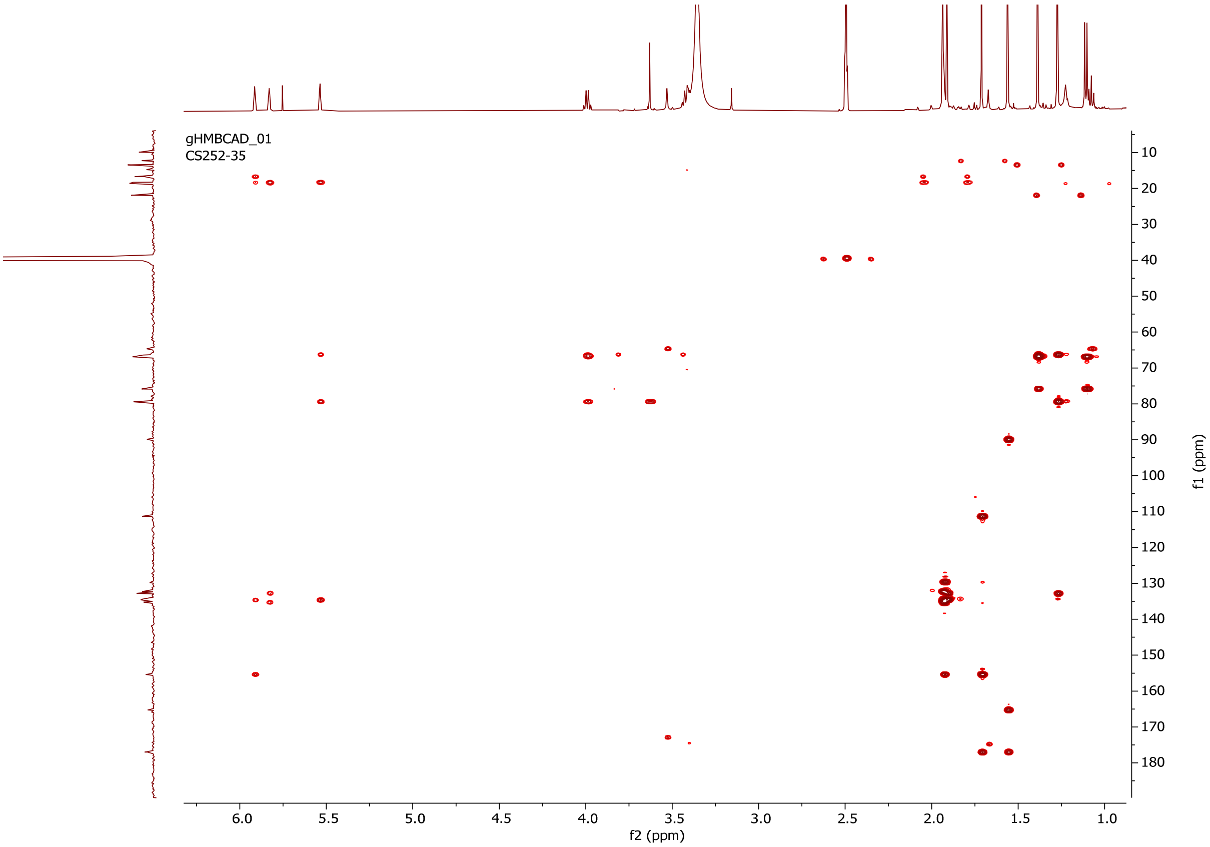

Figure S41. NOESY spectrum of compound **6**.

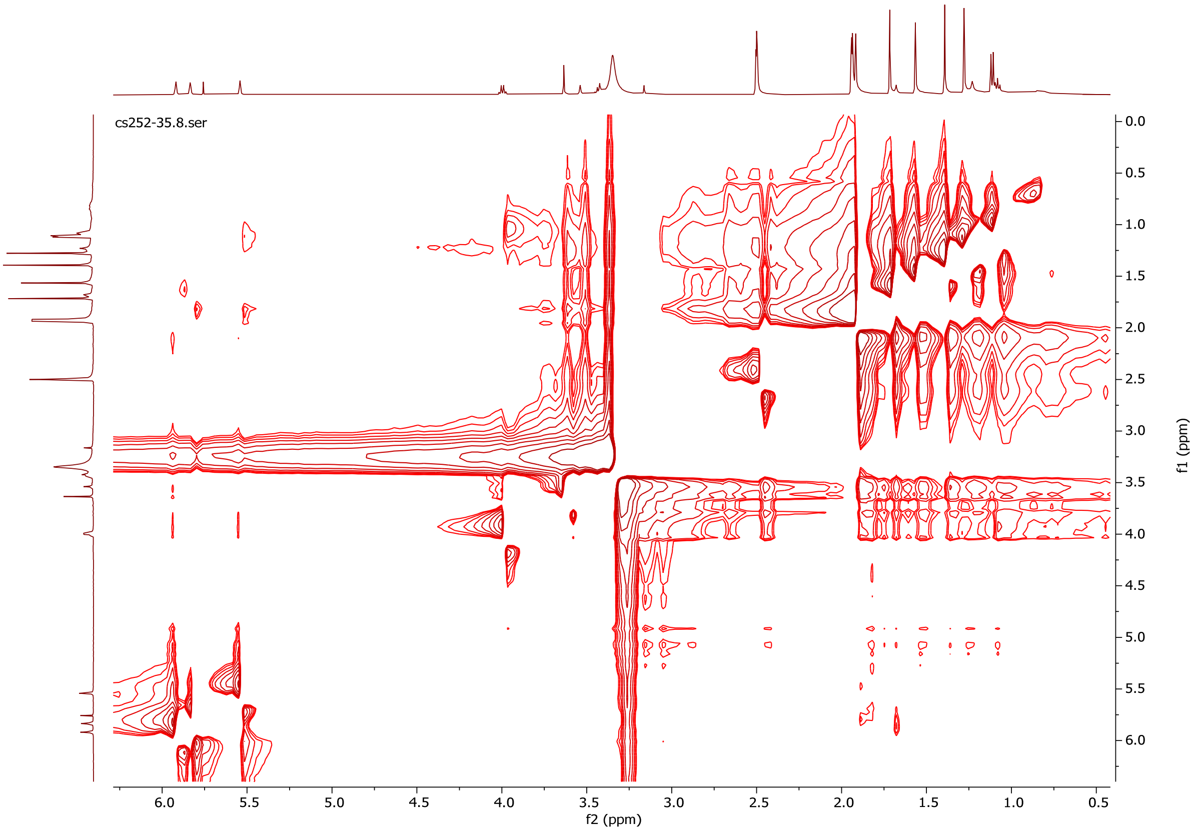

Figure S42. DP4+ probability Excel sheets of compound **4**.

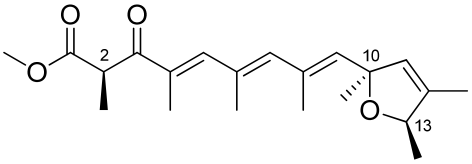

Isomer 1: 4a-(2*R*,10*S*,13*R*)

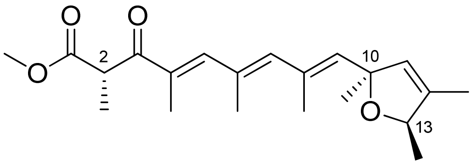

Isomer 2: 4b-(2*S*,10*S*,13*R*)

Functional	Solvent?	
mPW1PW91	PCM	
	Isomer 1	Isomer 2
sDP4+ (H data)	63.23%	36.77%
sDP4+ (C data)	63.30%	36.70%
sDP4+ (all data)	74.79%	25.21%
uDP4+ (H data)	97.06%	2.94%
uDP4+ (C data)	91.61%	8.39%
uDP4+ (all data)	99.72%	0.28%
DP4+ (H data)	98.27%	1.73%
DP4+ (C data)	94.96%	5.04%
DP4+ (all data)	99.91%	0.09%

Figure S43. DP4+ probability Excel sheets of compound **5**.

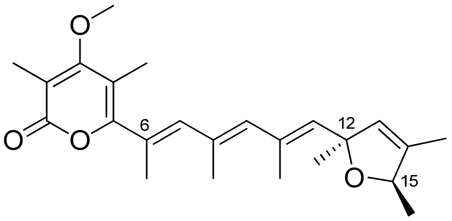

isomer 1: 5a-(6*E*,12*S*,15*R*)

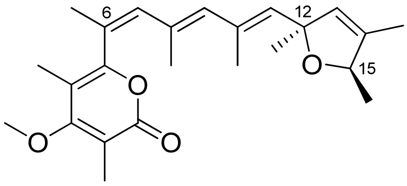

isomer 2: 5b-(6*Z*,12*S*,15*R*)

	Isomer 1	Isomer 2
sDP4+ (H data)	2.27%	97.73%
sDP4+ (C data)	0.03%	99.97%
sDP4+ (all data)	0.00%	100.00%
uDP4+ (H data)	0.01%	99.99%
uDP4+ (C data)	0.02%	99.98%
uDP4+ (all data)	0.00%	100.00%
DP4+ (H data)	0.00%	100.00%
DP4+ (C data)	0.00%	100.00%
DP4+ (all data)	0.00%	100.00%
